# Comparative membrane lipidomics of hepatocellular carcinoma cells reveals diacylglycerol and ceramide as key regulators of Wnt/β‐catenin signaling and tumor growth

**DOI:** 10.1002/1878-0261.13520

**Published:** 2023-09-20

**Authors:** Yagmur Azbazdar, Yeliz Demirci, Guillaume Heger, Dogac Ipekgil, Mustafa Karabicici, Gunes Ozhan

**Affiliations:** ^1^ Izmir Biomedicine and Genome Center (IBG) Dokuz Eylul University Health Campus Izmir Turkey; ^2^ Izmir International Biomedicine and Genome Institute (IBG‐Izmir) Dokuz Eylul University Izmir Turkey; ^3^ École Centrale de Nantes France; ^4^ Present address: Department of Biological Chemistry University of California Los Angeles CA USA; ^5^ Present address: Wellcome Sanger Institute Cambridge UK; ^6^ Present address: Board of Governors Regenerative Medicine Institute Cedars‐Sinai Medical Center Los Angeles CA USA; ^7^ Present address: Department of Molecular Biology and Genetics Izmir Institute of Technology Turkey

**Keywords:** ceramide, comparative lipidome profiling, diacylglycerol, hepatocellular carcinoma, plasma membrane, Wnt/β‐catenin pathway

## Abstract

Hepatocellular carcinoma (HCC) is largely associated with aberrant activation of Wnt/β‐catenin signaling. Nevertheless, how membrane lipid composition is altered in HCC cells with abnormal Wnt signaling remains elusive. Here, by exploiting comprehensive lipidome profiling, we unravel the membrane lipid composition of six different HCC cell lines with mutations in components of Wnt/β‐catenin signaling, leading to differences in their endogenous signaling activity. Among the differentially regulated lipids are diacylglycerol (DAG) and ceramide, which were downregulated at the membrane of HCC cells after Wnt3a treatment. DAG and ceramide enhanced Wnt/β‐catenin signaling by inducing caveolin‐mediated endocytosis of the canonical Wnt‐receptor complex, while their depletion suppressed the signaling activity along with a reduction of caveolin‐mediated endocytosis in SNU475 and HepG2 cells. Moreover, depletion of DAG and ceramide significantly impeded the proliferation, tumor growth, and *in vivo* migration capacity of SNU475 and HepG2 cells. This study, by pioneering plasma membrane lipidome profiling in HCC cells, exhibits the remarkable potential of lipids to correct dysregulated signaling pathways in cancer and stop abnormal tumor growth.

## Graphical abstract

Diacylglycerol and ceramide promote canonical Wnt signaling activity and caveolae‐mediated internalization of the Wnt‐receptor complex in hepatocellular carcinoma cells. On the other hand, inhibition of these lipids with diacylglycerol kinase (DGK) or myriocin, respectively, reduces canonical Wnt signaling activity, caveolae‐mediated internalization, spheroid area, and cell migration.

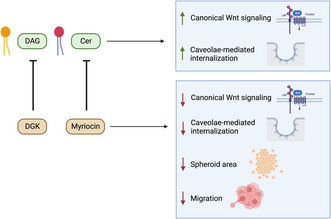

**Abbreviations**
APC: adenomatous polyposis coli
CE: cholesterol ester
Cer: ceramide
Ck1α: casein kinase 1α
CL: cardiolipin
CM: conditioned media
DAG: diacylglycerol
DAPI: 4′,6‐diamidino‐2‐phenylindole
DGK: diacylglycerol kinase
DGKA: diacylglycerol kinase alpha
Dkk1: dickkopf 1
DMEM: Dulbecco's Modified Eagle Medium
Down: downregulated
dpf: days post‐fertilization
DRL: differentially regulated lipid
Gsk3β: glycogen synthase kinase 3β
HCC: hepatocellular carcinoma
HexCer: hexosylceramide
LiCl: lithium chloride
LPA: lyso‐phosphatidate
LPC: lysophosphatidylcholine
LPE: lysophosphatidylethanolamine
LPG: lysophosphatidylglycerol
LPI: lysophosphatidylinositol
LPS: lysophosphatidylserine
Lrp5/6: low‐density lipoprotein‐receptor‐related protein 5/6
PA: phosphatidate
pBAR: β‐catenin‐activated reporter
PC: phosphatidylcholine
PCA: principal component analysis
PE: phosphatidylethanolamine
PFA: paraformaldehyde
PG: phosphatidylglycerol
PI: phosphatidylinositol
PP2A: protein phosphatase 2A
PS: phosphatidylserine
RLuc: renilla luciferase reporter
SM: sphingomyelin
TAG: triacylglycerol
Tcf/Lef: T‐cell factor/lymphoid enhancer factor
Up: upregulated

## 1 Introduction

Hepatocellular carcinoma (HCC) is the most common form of all primary liver cancers in adults and the third most common cause of cancer‐related deaths worldwide. The multistage process of HCC development based on the accumulation of several mutations that promote malignant transformation and tumor growth is only partially understood. Nevertheless, the Wnt/β‐catenin signaling pathway is known to be extensively involved in the initiation and progression of HCC [1]. Owing to its key regulatory roles in development and homeostasis, Wnt signaling has been linked to cancer since the discovery that activation of *int1* (*Wnt1*) resulted in mammary hyperplasia and tumors [2, 3, 4]. Wnt/β‐catenin signaling is highly activated in approximately 50% of HCC cases [5, 6]. Excessive Wnt signaling activation has been reported in several subgroups of HCC, that is, the “Wnt‐TGFβ subclass,” the “progenitor subclass” with mutations in *AXIN1*, and the relatively more heterogeneous “non‐proliferation class” with mutations in the β‐catenin gene *CTNNB1* [7, 8]. Aberrant Wnt signaling activity in HCC has been associated with enhanced tumor growth, immune escape, and resistance to therapy [5, 9, 10, 11, 12].

Wnt/β‐catenin (canonical Wnt) signaling is a key signaling pathway that controls cell fate determination during embryonic development, maintenance of tissue homeostasis, and regeneration [13, 14, 15]. Wnt/β‐catenin pathway is held in the inactive state in the absence of a canonical Wnt ligand, leading to phosphorylation and degradation of β‐catenin by a cytoplasmic multiprotein complex, termed the β‐catenin destruction complex, consisting of casein kinase 1α (Ck1α), glycogen synthase kinase 3β (Gsk3β), the tumor suppressors adenomatous polyposis coli (APC) and Axin, protein phosphatase 2A (PP2A) and the E3‐ubiquitin ligase β‐transducin repeats‐containing protein β‐TrCP [16]. Signal transduction is initiated by the interaction of the canonical Wnt ligand with the receptor Frizzled (Fz) and the coreceptor low‐density lipoprotein‐receptor‐related protein 5/6 (Lrp5/6) preferentially in the ordered membrane nanodomains [17]. Next, components of the β‐catenin destruction complex are recruited to the Wnt‐receptor complex at the plasma membrane, leading to phosphorylation of Lrp5/6 by Ck1α and Gsk3β, allowing for accumulation of β‐catenin in the cytoplasm and its subsequent translocation into the nucleus, where β‐catenin binds to the T‐cell factor/lymphoid enhancer factor (Tcf/Lef) family of transcription factors to initiate the expression of target genes [18].

The canonical Wnt signaling pathway has been widely studied in human diseases, especially cancer, concerning its plasma membrane components including the ligands, receptors, coreceptors, and secreted or membrane‐bound pathway modulators [19, 20, 21, 22]. The plasma membranes are dynamic and heterogeneous fluidic structures consisting of proteins and lipids that move via lateral, rotational, and transverse diffusion, and they separate the cell from the extracellular environment [23]. The membrane is further compartmentalized into numerous micro‐ or nanodomains that enable specialized cellular functions via regulation of receptor trafficking and downstream signaling [24]. The compartmentalization can be disturbed due to mutations in genes encoding membrane proteins, abnormalities in membrane structural organization, or misassemble of membrane proteins and lipids, leading to disruption of cell signaling networks and promotion of carcinogenic activities. The plasma membranes of tumor cells display notable alterations in their composition, structural organization, and functional properties. Structural alterations in the membrane have also been associated with cellular multidrug resistance in cancer that may occur due to poor transport of drugs across the membrane and active efflux of therapeutic molecules from the cytoplasm to the extracellular environment via membrane transporters [25]. Thus, it is rational to expect a significant difference in the membrane lipidome profiles of healthy and cancer cells [26, 27].

While membrane lipid content has been proposed to vary between cancer cells and healthy cells [28, 29, 30, 31], membrane lipidome profiles of cancer cells, in particular in a variety of HCC cell lines with varying canonical Wnt signaling activities, have not been identified so far. This approach is especially important to test whether cancer cells can be distinguished from healthy cells concerning their membrane lipid composition. Here, we postulate that the membrane lipidome profiles of several HCC cell lines with different endogenous Wnt signaling activities differ significantly from each other and the healthy control liver cells. To address this assumption, we took advantage of advanced shotgun lipidomics to unravel the lipids in the plasma membranes isolated from six different HCC cell lines. Initially, we validated that these HCC cells have different levels of Wnt/β‐catenin signaling activity and respond differentially to Wnt pathway activation or inhibition. By globally comparing the membrane lipidome profiles of these cells, we have unraveled that their membrane lipid compositions were significantly different from each other and the healthy liver cells. Moreover, activation or inhibition of the Wnt pathway in HCC cells resulted in further differentiation of their membrane lipidome profiles for main lipid categories and lipid species. Among those were particular lipids that were not only differentially regulated in HCC cells but also responsive to Wnt pathway manipulation. Next, to examine whether the lipids that are differentially regulated in response to Wnt pathway manipulation can be exploited to stop tumorigenic characteristics of HCC cells, we selected the lipids diacylglycerol (DAG) and ceramide, which were both downregulated at the membrane of HCC cells after canonical Wnt pathway activation. DAG and ceramide treatment potently activated Wnt/β‐catenin signaling by enhancing caveolin‐mediated endocytosis of the canonical Wnt‐receptor complex, while their depletion significantly reduced the signaling activity and caveolin‐mediated endocytosis in SNU475 and HepG2 cells. Depletion of DAG and ceramide suppressed the proliferative capacity, tumor spheroid growth, and migration of HCC cells. Overall, by unraveling the membrane lipidome profiles of HCC cells in response to manipulation of Wnt/β‐catenin signaling, our study pioneers testing the potential of cell‐specific lipid fingerprint to stop tumor progression.

## 2 Materials and methods

### 2.1 Cell culture and conditioned media production

HUH7 (RRID: CVCL_0336), SNU475 (RRID: CVCL_0497), Hep3B (Hep3B2.1‐7, RRID: CVCL_0326), HepG2 (RRID: CVCL_0027), SNU398 (RRID: CVCL_0077), and Mahlavu cells (RRID: CVCL_0405) were cultured in RPMI 1640 media supplemented with 10% fetal bovine serum (FBS) at 37 °C in 5% (v/v) CO_2_ humidified environment. THLE2 (RRID: CVCL_3803) cells were grown in a bronchial epithelial cell growth medium bullet kit (Lonza, Basel, Switzerland) with 10% FBS at 37 °C in 5% (v/v) CO_2_. HEK293T cells were cultured in high glucose Dulbecco's Modified Eagle Medium (DMEM) supplemented with 10% FBS. The cell lines were a gift from Prof. Mehmet Ozturk (HUH7 and Mahlavu; Izmir Tinaztepe University, Izmir, Türkiye), Prof. Esra Erdal (SNU398 and SNU475; Izmir Biomedicine and Genome Center, Izmir, Türkiye), and Prof. Nese Atabey (Hep3B, HepG2, and THLE2; Izmir Tinaztepe University, Izmir, Türkiye). The authentication of the cell lines was achieved by DNA profiling at the University of Colorado Cancer Center (UCCC) DNA Sequencing & Analysis Core (CO, USA) using AmpFLSTR™ Identifiler™ PCR Amplification Kit (Thermo Fisher Scientific, MA, USA). All cell lines were tested for mycoplasma contamination using the MycoAlert® Mycoplasma Detection Kit (Lonza, Basel, Switzerland) and confirmed negative prior to cryopreservation and establishment of a cell stock. Wnt3a was produced in murine L Wnt‐3a cells (ATCC, VA, USA). L Wnt‐3a cells were grown in high glucose DMEM supplemented with 10% FBS in a 100‐mm tissue culture plate. When cells were 80–90% confluent, they were split into new 100‐mm plates. Wnt3a‐conditioned media (CM) were collected on the 2nd, 4th, and 6th days after reaching 100% confluence and stored at 4 °C until use. The secreted canonical Wnt inhibitor dickkopf 1 (Dkk1) was produced from HEK293T cells that were seeded in a 100‐mm tissue culture plate. The next day, cells were transfected with 5 μg of pCS2P + dkk1GFP plasmid using Lipofectamine 2000 (Thermo Fisher Scientific, MA, USA). Dkk1GFP CM was collected on the 2nd, 4th, and 6th days after reaching 100% confluence and stored at 4 °C.

### 2.2 Transfection, treatment of cells with conditioned media, and luciferase assay

Cells were seeded on 24‐well cell culture plates and transfected in triplicates with 20 ng of firefly luciferase reporter pGL3 β‐catenin‐activated reporter (pBAR) (19099249) and 5 ng of renilla luciferase reporter pGL4.73 hRLuc/SV40 (RLuc; Promega, WI, USA) and a membrane GFP (75 ng as the control for transfection) or a plasmid containing diacylglycerol kinase alpha (DGKA, Addgene plasmid #35404, [32]) using FuGENE HD transfection reagent (1 μg DNA/1 μL) (Promega, WI, USA). Twenty‐four hours after the transfection, cells were treated with 500 μL of Wnt3a or Dkk1GFP CM for 16 h or 40 mm of lithium chloride (LiCl) for 3 h. Reporter activity was measured using the dual luciferase reporter assay kit (Promega, WI, USA). Statistical significance analysis was conducted using Student's *t*‐test. ****P < 0.0001, ***P < 0.001, **P < 0.01, and *P < 0.05. Error bars represent standard deviation (SD).

### 2.3 Isolation of giant plasma membrane vesicles and lipidomics assay

Cells were seeded in 100 mm Petri dishes in the appropriate media stated above, cultured to approximately 80% confluence, and incubated with control, Wnt3a, or Dkk1 CM for 1 h at 37 °C. Afterward, cells were treated with 2 mm N‐ethyl maleimide in 5 mL of GMPV isolation buffer (2 mm CaCl_2_, 150 mm NaCl, 10 mm HEPES, pH 7.4) for 4–5 h at 37 °C [33, 34]. The supernatant was transferred to a new tube and spun at 100 ***g*** for 3 min. The supernatant of 4.5 mL was split into tubes and spun at 17 000 ***g*** for 75 min at 4 °C. The pellet was resuspended in 100 μL of 150 mm ammonium bicarbonate. Ten ug of the sample was sent for lipidomics analysis to Lipotype GmbH (Dresden, Germany).

### 2.4 Lipid extraction for mass spectrometry lipidomics

Mass spectrometry‐based lipid analysis was performed by Lipotype GmbH (Dresden, Germany) as described [35]. Lipids were extracted using a two‐step chloroform/methanol procedure [36]. Samples were spiked with internal lipid standard mixture containing: cardiolipin 16 : 1/15 : 0/15 : 0/15 : 0 (CL), ceramide 18 : 1;2/17 : 0 (Cer), cholesterol ester 20 : 0 (CE), DAG 17 : 0/17 : 0, hexosylceramide 18 : 1;2/12 : 0 (HexCer), lyso‐phosphatidate 17 : 0 (LPA), lysophosphatidylcholine 12 : 0 (LPC), lysophosphatidylethanolamine 17 : 1 (LPE), lysophosphatidylglycerol 17 : 1 (LPG), lysophosphatidylinositol 17 : 1 (LPI), lysophosphatidylserine 17 : 1 (LPS), phosphatidate 17 : 0/17 : 0 (PA), phosphatidylcholine 17 : 0/17 : 0 (PC), phosphatidylethanolamine 17 : 0/17 : 0 (PE), phosphatidylglycerol 17 : 0/17 : 0 (PG), phosphatidylinositol 16 : 0/16 : 0 (PI), phosphatidylserine 17 : 0/17 : 0 (PS), sphingomyelin 18 : 1;2/12 : 0;0 (SM), triacylglycerol 17 : 0/17 : 0/17 : 0 (TAG). After extraction, the organic phase was transferred to an infusion plate and dried in a speed vacuum concentrator. 1st step dry extract was re‐suspended in 7.5 mm ammonium acetate in chloroform/methanol/propanol (1 : 2 : 4, v : v : v) and 2nd step dry extract in 33% ethanol solution of methylamine/chloroform/methanol (0.003 : 5 : 1; v : v : v). All liquid handling steps were performed using Hamilton Robotics STARlet robotic platform with the Anti Droplet Control feature for organic solvent pipetting.

### 2.5 MS data acquisition

Samples were analyzed by direct infusion on a QExactive mass spectrometer (Thermo Fisher Scientific, MA, USA) equipped with a TriVersa NanoMate ion source (Advion, NY, USA). Samples were analyzed in both positive and negative ion modes with a resolution of Rm/z = 200 = 280 000 for MS and Rm/z = 200 = 17 500 for MSMS experiments, in a single acquisition. MSMS was triggered by an inclusion list encompassing corresponding MS mass ranges scanned in 1 Da increments [37]. Both MS and MSMS data were combined to monitor CE, DAG, and TAG ions as ammonium adducts; PC, PC O‐, as acetate adducts; and CL, PA, PE, PE O‐, PG, PI, and PS as deprotonated anions. MS only was used to monitor LPA, LPE, LPE O‐, LPI, and LPS as deprotonated anions; Cer, HexCer, SM, LPC, and LPC O‐ as acetate adducts.

### 2.6 Data analysis and post‐processing

Data were analyzed with in‐house developed lipid identification software based on lipidxplorer [38, 39]. Data post‐processing and normalization were performed using an in‐house developed data management system. Only lipid identifications with a signal‐to‐noise ratio of >5 and a signal intensity fivefold higher than in corresponding blank samples were considered for further data analysis.

### 2.7 Bioinformatics analysis

The raw lipid concentrations (in pmol) were used in data analysis. These were normalized by the sum of concentrations of all lipid species within each sample and averaged across replicates to calculate concentration fold change between all cancer cell lines and healthy control cells and between treated and untreated cells of each cell line. The significance of differential regulation for each lipid was assessed by a two‐tailed Student's *t*‐test between normalized concentrations. Missing values (i.e., non‐measurable concentrations below the detection limit) were neglected in calculations, and only lipids with at least two replicates in both conditions were considered in each contrast. Statistical significance was called at *P*‐value < 0.05. Differential regulation contrasts and lipids were clustered by Pearson correlation of log2 fold change values (missing values and non‐significant effects were replaced by zero) and shown on a heatmap. Relative concentrations (in mol%/sample) were used in the principal component analysis (PCA). Data were plotted using the packages ggplot2 (3.3.2), pheatmap (1.0.12), ComplexHeatmap (2.4.2), and UpSetR (1.4.0) [40, 41, 42, 43].

### 2.8 Treatment of cells with lipids and lipid synthesis inhibitors

Cells were seeded on 24‐well culture plates and transfected in triplicates with 20 ng of pBAR, 5 ng of RLuc, and a membrane GFP using the FuGENE HD transfection reagent as described above. Twenty‐four hours after transfection, the chemical/enzyme was added to the SNU475 cells at the concentrations indicated as follows: DAG 16 : 0‐18 : 1 DG‐ 1‐palmitoyl‐2‐oleoyl‐sn‐glycerol, 50 μg·mL^−1^, Avanti polar lipids, AL, USA, ceramide (C18 Ceramide (d18 : 1/18 : 0)‐ N‐stearoyl‐D‐erythro‐sphingosine, 25 μg·mL^−1^, Avanti polar lipids, AL, USA), DGKA, 0.5 μg·mL^−1^ MyBioSource, CA, USA) or myriocin (12.5 μm, Cayman Chemical Company, Michigan, USA). For HepG2 cells, the concentrations of the chemical/enzyme were as follows: DAG 200 μg·mL^−1^, ceramide 100 μg·mL^−1^, DGK 0.5 μg·mL^−1^, and myriocin 12.5 μm. SNU475 and HepG2 cells were incubated at 37 °C for 4 h with DAG, ceramide, and DGK and 48 h with myriocin, and treated with Wnt3a or Dkk1 CM containing the drugs for 16 h. Reporter activity was measured using a dual luciferase reporter assay kit and significance was tested using Student's *t*‐test as described above.

### 2.9 Immunofluorescence staining

HeLa cells were seeded on cover glass in a 24‐well tissue culture plate and transfected with a DGKA‐containing plasmid as described above. HEK293, SNU475, and HepG2 were likewise seeded on cover glass and treated with DAG, ceramide, and DGK for 4 h or myriocin for 48 h at the concentrations stated in the previous sub‐section. Next, control CM, Wnt3a CM, or Dkk1 CM was added into plates and incubated overnight at 37 °C in a 5% (v/v) CO_2_ humidified environment. For immunostaining, cover glasses were removed and cells were fixed with 4% paraformaldehyde (PFA) in PBS for 15 min. They were washed three times with PBS for 5 min each, PBS including 0.5% Tween‐20 for 10 min, three times with PBS for 5 min each and incubated with blocking buffer (90% PBS, 1% goat serum, 0.1% Tween 20, 1% BSA, 2.25% glycine) for 30 min. Next, cells were incubated with the primary antibodies rabbit anti‐phospho‐β catenin (Ser675, 1 : 100, D2F1, Cell Signaling Technology, MA, USA), mouse anti‐β‐catenin (1 : 100, 12F7, ab22656, Abcam, MA, USA), rabbit Biotin‐conjugated anti‐DAG (1 : 500, MBS2013372, MyBioSource Inc., CA, USA), and rabbit anti‐caveolin‐1 (1 : 500, 3238S, Cell Signaling Technology, MA, USA) in PBS containing 1% BSA and 0.3% Triton‐X overnight at 4 °C. The next day, cells were washed three times with PBS for 5 min each, incubated with the secondary antibodies goat anti‐mouse IgG, super‐clonal recombinant secondary antibody, Alexa Fluor 488 (1 : 800, ThermoFisher Scientific, MA, USA) and goat anti‐Rabbit IgG cross‐adsorbed secondary antibody, Alexa Fluor 594 (1 : 800, ThermoFisher Scientific, MA, USA) for 2 h at room temperature (RT). Next, cells were washed five times with PBS for 5 min each, their nuclei were counterstained with 4′,6‐diamidino‐2‐phenylindole (DAPI, 0.5 μg·mL^−1^, 4083S, Cell Signaling Technology, MA, USA) and imaged by using an LSM 880 laser scanning confocal microscope (Carl Zeiss AG, Oberkochen, Germany) or Observer Z1 Inverted Microscope with Apotome (Carl Zeiss AG, Oberkochen, Germany).

### 2.10 Diacylglycerol measurement assay

HepG2 cells were treated with DGK, 0.5 μg·mL^−1^ MyBioSource, CA, USA) for 4 h. Next, lipid content isolation and determination of DAG content were performed according to manufacturer's instructions (ab242293, Abcam, MA, USA). Significance was tested using Student's *t*‐test.

### 2.11 Western blotting

Cells were lysed with RIPA buffer. Protein samples were prepared in 5X loading dye and separated by SDS gel electrophoresis by running on 10% acrylamide‐bisacrylamide gel. Proteins were transferred to polyvinylidene fluoride membrane (GE Healthcare Life science, IL, USA) and membrane was blocked in 5% milk powder for 45 min at RT. Next, membrane blot was incubated overnight at 4 °C with the following primary antibodies: rabbit anti‐phospho‐β catenin (Ser675, 1 : 1000, D2F1, Cell Signaling Technology, MA, USA), mouse anti‐β‐catenin (1 : 1000, 12F7, ab22656, Abcam, MA, USA) and mouse anti‐caveolin (1 : 500, 7C8): sc‐53564, Santa Cruz Biotechnology Inc., TX, USA), rabbit anti‐β‐Actin (1 : 4000; 8457S, Cell Signaling Technology, MA, USA). The next day, blot was washed with TBST three times and incubated with the secondary antibodies goat anti‐rabbit IgG (H + L), DyLight™ 800 4X PEG (1 : 2500, SA5‐35571, Thermo Fischer, MA, USA) or Cy5‐conjugated AffiniPure donkey anti‐mouse IgG (H + L) (1 : 2500, 715‐175‐150, Jackson ImmunoResearch, PA, USA).

### 2.12 Colony formation assay

SNU475 and HepG2 cells were seeded as 1000 cells/well in a 6‐well culture plate and let grow into colonies for 8 days. After removal of the supernatant, methanol was added to the cells and kept at −20 °C for 10 min. Next, cells were stained with crystal violet (0.5% w/v in distilled water) for 20 min at RT and rinsed several times with distilled water. Plates were left to dry in the incubator and imaged under a light microscope. The number of colonies was calculated by the imagej Fiji cell counter plugin (ImageJ, US National Institutes of Health, Bethesda, Maryland, USA). Significance was tested using Student's *t*‐test.

### 2.13 Spheroid formation assay

The hanging drop method was used for this assay. Droplets of 30 μL cell culture media containing 1000 cells were pipetted on the interior of a 100 mm petri dish and the dish lid was inverted. Cells were incubated at 37 °C for 4 days until spheroid formation. Next, the media were replaced with media containing lipid synthesis inhibitors. After 24 h or 48 h, the cells were imaged under a stereomicroscope. Spheroid areas were calculated using imagej. Significance was tested using Student's *t*‐test.

### 2.14 Zebrafish xenograft assay

SNU475 and HepG2 cells were trypsinized in cell culture plates, washed with PBS, and incubated with 2 mg·mL^−1^ DiI in PBS at 37 °C for 20 min. After washing once with FBS and twice with PBS, cells were resuspended in 10% FBS in 1X PBS at a final density of 40 000 cells·μL^−1^. Wild‐type AB zebrafish larvae were obtained from Izmir Biomedicine and Genome Center Vivarium‐Zebrafish Core Facility and dechorionated at 2 days post‐fertilization (dpf) by incubating in 0.1 mg·mL^−1^ pronase (Sigma‐Aldrich, MO, USA) solution for 10 min at 28 °C. Larvae were then anesthetized with 1 mg·mL^−1^ Tricaine in E3 medium and transferred to a microinjection plate of 3% agarose in E3. Borosilicate glass capillaries with a length of 4 inches and an OD of 1.0 mm (World Precision Instruments, FL, USA) were used for microinjection. 200–300 SNU475 cells and 700–800 HepG2 cells were injected into the yolk sac of wild‐type AB zebrafish larvae. Larvae were incubated at 34 °C in fresh E3 medium overnight. The next day, larvae that showed infiltration of tumor cells into blood circulation were discarded. Larvae with injected cells in the yolk sac were kept and processed further for drug treatments. Zebrafish larval xenografts were fixed in 4% PFA in PBS at 6 dpf and 7 dpf for SNU475 cells and HepG2 cells, respectively. Xenografts were imaged and the percentage of micrometastasis was determined. Significance was tested using Student's *t*‐test.

### 2.15 Ethics statement

The animal study was reviewed and approved by the Animal Experiments Local Ethics Committee of Izmir Biomedicine and Genome Center (IBG‐AELEC) with the license number 14/2017.

## 3 Results

### 3.1 Different HCC cell lines vary in their activity of Wnt/β‐catenin signaling and plasma membrane lipid composition

**Figure 1:**
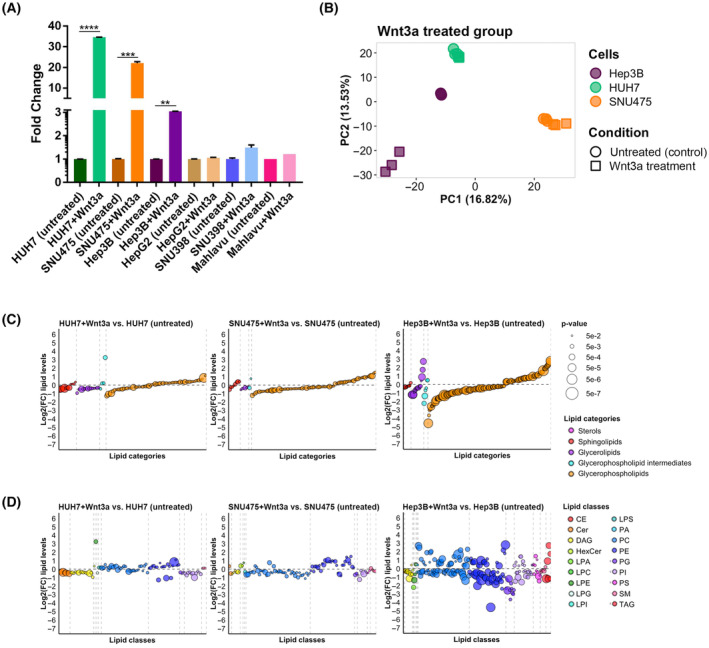
Different HCC cell lines vary in their activity of Wnt/β‐catenin signaling and plasma membrane lipid composition. (A) Comparison of canonical Wnt signaling activity in various HCC cell lines with known mutations in *β‐CATENIN*, *APC*, or *AXIN‐1* genes. Average and SD of the mean (error bars) values of pBAR luciferase reporter activity represent Wnt/β‐catenin signaling activity (normalized to renilla luciferase activity) in untreated and Wnt3a‐treated HUH7, SNU475, Hep3B, HepG2, SNU398, and Mahlavu cells. Statistical significance was evaluated using an unpaired *t*‐test. ****P < 0.0001, ***P < 0.001, and **P < 0.01. Error bars represent SD. (B) PCA of untreated and Wnt3a‐treated Hep3B, HUH7, and SNU475 cells. Cell lines are represented by different colors and treatment statuses by different shapes. Differential regulation plots of lipids arranged by (C) lipid categories and (D) lipid classes in response to Wnt3a stimulation in HUH7, SNU475, and Hep3B cell lines. Lipids are represented by dots arranged regularly on the *x*‐axis with their log2 fold change represented on the *y*‐axis. Dots are colored by (C) lipid category or (D) lipid class and sized proportionally to the statistical significance of differential regulation. Only DRL species are shown for each contrast. Vertical dashed lines separate lipid categories/classes. A horizontal dashed line separates up‐ and down‐regulated lipids. Three independent experiments were performed for (A) and three biological replicates (*n* = 3) were used in each experimental group for the analyses in (B–D).

The lipid compositions significantly vary between plasma membranes of different cell types, resulting in distinct membrane properties that in turn strongly influence the activity of membrane‐bound proteins and related signaling pathways. Understanding this interdependency necessitates a good knowledge of the lipid composition of the membranes. Thus, to examine the relationship between the lipid composition of the plasma membrane and regulation of Wnt/β‐catenin signaling activity, we aimed to exploit high‐end lipidomics technology to unravel the membrane lipidome profiles of six different HCC cell lines. Initially, we set out to determine the endogenous canonical Wnt signaling activities in different HCC cell lines, that is, HUH7, SNU475, Hep3B, HepG2, SNU398, and Mahlavu cells, all of which have mutations in at least one component (*β‐CATENIN*, *APC*, or *AXIN‐1*) of Wnt/β‐catenin signaling, along with the control HEK293T cells. According to the activation of the pBAR reporter of Tcf/Lef‐mediated transcription, Mahlavu cells exhibited the lowest signaling activity while HepG2 and SNU398 cells had the highest activity (Fig. S1A). Next, to test the response of the HCC cells to manipulation of Wnt signaling, we treated the cells with the CM of the canonical ligand Wnt3a or the canonical Wnt inhibitor Dkk1. Wnt3a was able to activate Wnt signaling in HUH7, SNU475, and Hep3B cells, showing that Wnt signaling can be exogenously induced in these cells (Fig. 1A). In contrast, Dkk1 treatment could significantly suppress Wnt signaling in all five HCC cell lines (Fig. S1B). Thus, to perform lipid fingerprinting we collected giant plasma membrane vesicles from the cells and formed three experimental groups: (a) Non‐treated group: HUH7, SNU475, Hep3B, HepG2, SNU398, Mahlavu cells compared to the healthy control hepatocyte cells THLE2. (b) Wnt3a‐treated group: HUH7 + Wnt3a, SNU475 + Wnt3a and Hep3B + Wnt3a cells compared to HUH7, SNU475, and Hep3B cells. (c) Dkk1‐treated group: HepG2 + Dkk1 and SNU398 + Dkk1 cells compared to HepG2 and SNU398 cells. Following lipid extraction and mass spectrometry‐based lipid analysis performed by Lipotype GmbH, we performed PCA on the three experimental groups (Fig. 1B; Fig. S1B,D). Individual samples of untreated HCC cells were well separated from each other and the control samples (Fig. S1B). There was a remarkable variance between healthy control positioned on one side and all untreated HCC cells on the other side. In particular, SNU398 and SNU475 stood out from the other cells. PCA revealed that treatment of HCC cells with Wnt3a or Dkk1 resulted in changes in their lipid composition and significant separation from each other. Wnt3a treatment resulted in minor changes in the membrane lipidome of HUH7 and SNU475 cells, whereas strong variance was detectable in Hep3B cells induced with Wnt3a (Fig. 1C). Dkk1 treatment resulted in significant changes in the membrane lipidome in both HepG2 and SNU398 cells (Fig. S1D). Together, these results indicate that HCC cells with different levels of endogenous Wnt/β‐catenin activity differ in their response to Wnt pathway manipulation and that this differential response of HCC cells is reflected in their membrane lipid composition.

### 3.2 Plasma membranes of different HCC cells diverge concerning the distribution of main lipid categories and lipid species

To understand how plasma membrane lipidome profiles alter between different HCC cells, we plotted the log2 fold change in the lipids that were detected in the steady state by mass spectrometry in six different HCC cells as compared to the healthy control liver (THLE2) cells (Fig. S2A,B). A great number of glycerophospholipids were differentially regulated in all untreated HCC cell types as compared to the healthy control cells (Fig. S2A). Glycerophospholipid intermediates were mostly upregulated (Up) in HCC cells except for SNU475 and Mahlavu cells. Sterols, sphingolipids, and glycerolipids were not uniformly altered in one specific direction between HCC and healthy control cells. Next, we examined the differential regulation patterns for the alterations in the following lipid classes: CEs, Cer, CL, DAG, HexCer, LPA, LPC, LPE, LPG, LPI, LPS, PA, PC, PE, PG, PI, PS, SM, and TAG. When examined in terms of lipid classes, CE, Cer, HexCer, and SM (except for Mahlavu cells) were generally downregulated (Down) and LPA, LPC, LPE, LPI, LPS, PI, and PS were generally Up in HCC cells lines compared to the healthy control cells (Fig. S2B). Notably, most PE class lipids were Down in SNU398 but Up in HUH7, and several lipids of this class were dramatically Down in Hep3B and Mahlavu cells. In addition, a vast majority of the lipids in the PG and PI classes were Up in all HCC cell lines. Overall, consistently with the PCA, differential regulation of lipids is most remarkable in Hep3B, SNU398, and Mahlavu cells concerning the lipid numbers and statistical significance.

### 3.3 Membrane lipids of HCC cells alter significantly in response to activation or inhibition of Wnt/β‐catenin signaling

When compared with control hepatocyte cells, HCC cells carrying mutations in Wnt pathway components have distinctive membrane lipidome profiles. Thus, we next aimed at testing whether modulation of Wnt/β‐catenin signaling in HCC cells affects their membrane lipids. First, we compared the lipidome profiles of the HCC cells that respond to Wnt3a treatment to those of the untreated cells by plotting the log2 fold changes of the lipids that are differentially regulated after treatment (Fig. 1C,D, Table S1). Wnt3a induction had a milder effect on all lipid categories in Huh7 and SNU475 cells, while it resulted in strong differential regulation, and more prominently downregulation, of the lipids in Hep3B cells (Fig. 1C, Table S1). In HUH7 and SNU475 cells treated with Wnt3a, Cer, DAG, PG, and PI classes of lipids were mostly Down, whereas most lipids in the PE class were Up as compared to their untreated counterparts (Fig. 1D, Table S1). PC class of lipids appeared to be regulated mildly and oppositely in HUH7 + Wnt3a and SNU475 + Wnt3a cells. In Hep3B + Wnt3a cells, the number of differentially regulated lipids (DRLs) was much higher and most lipids in the Cer, DAG, and LPC classes were more strongly Down. The majority of the lipids in PC, PE, PG, PI, PS, and SM classes were likewise Down in Hep3B + Wnt3a cells. Overall, DAG and Cer lipid classes decreased dramatically in the plasma membrane after Wnt3a treatment in all cell lines.

Next, we tested how the membrane lipids change in HCC cells upon inhibition of Wnt/β‐catenin signaling. Dkk1 treatment resulted in differential regulation of a larger number of lipids in HepG2 cells than in SNU398 cells (Fig. S3A,B, Table S1). In HepG2 + Dkk1 cells, the majority of the DRLs, including the glycerophospholipid intermediates, was Down. Interestingly, sphingolipids and glycerolipids were unanimously Down in SNU398 + Dkk1 cells while glycerolipids were almost equally distributed between Up and Down (Fig. S3A, Table S1). While lipids of the DAG and SM classes were mostly Up in HepG2 + Dkk1 cells, they were uniformly Down in SNU398 + Dkk1 cells (Fig. S3B, Table S1). The Cer class of lipids was Down in both HepG2 + Dkk1 and SNU398 + Dkk1 cells. Although the PC class of lipids was overall Down in both cell types after Dkk1 treatment, some members of this class displayed relatively strong upregulation signals in SNU398 + Dkk1 cells. Moreover, lipids in LPA, LPC, LPE, and LPI classes showed differential regulation only in HepG2 + Dkk1 cells where the majority of these lipids were Down. Thus, activation or inhibition of Wnt/β‐catenin signaling causes alteration of plasma membrane lipids in HCC cells. Interestingly, treatment with Wnt3a or Dkk1 did not exert exactly opposite effects on the membrane lipid composition of HCC cells, most likely due to distinct receptor internalization pathways activated by Wnt3a and Dkk1 [44]. Thus, we performed the functional analyses by using the membrane lipids that altered consistently in response to Wnt3a treatment.

### 3.4 Global comparison of membrane lipidome profiles reveals differential regulation of lipids in HCC cells and healthy cells

We next aimed to unravel the relationship between the altered lipidome profiles and the cancer cells using bioinformatic tools. To identify global and specific trends across different HCC cell lines concerning the regulation of lipid classes and categories at the plasma membrane, we pooled all DRLs obtained in comparisons of Wnt3a/Dkk1‐treated HCC cells to untreated HCC cells as well as of untreated or Wnt3a/Dkk1‐treated HCC cells to healthy control cells. The generated heatmap summarizes differential regulation profiles of the lipids clustered based on log2 fold changes (Fig. S4A). In the heatmap, we identified two main clusters of lipid regulation profiles. First, the horizontal clustering of the heatmap showed that the rate of change in lipids observed in HCC cells in response to Wnt pathway manipulation (activation with Wnt3a or inhibition with Dkk1) was much lower than the rate of change in lipids observed in HCC cells compared to healthy cells, independent of manipulation. Because of the distinct rates of change, lipids were clustered mainly based on the cancer effects rather than the Wnt pathway manipulation effects. In other words, the global influence of “cancer” on the membrane lipidome is greater than that of “Wnt pathway manipulation.” Second, according to the vertical clustering of the heatmap, we identified five main patterns of DRLs: (a) Lipids that were generally Up in HCC cells (with or without Wnt pathway manipulation) as compared to healthy control cells and weakly responsive (mostly as downregulation) to Wnt pathway manipulation, particularly to Wnt3a. (b) Lipids that were generally Down in HCC (with or without Wnt pathway manipulation) as compared to healthy control cells and weakly responsive to Wnt pathway manipulation. (c) Lipids that were specifically Up in SNU398 and SNU475 cells (with or without Wnt pathway manipulation) as compared to healthy control cells. (d) Lipids that were specifically Down in SNU398 and SNU475 cells (with or without Wnt pathway manipulation) as compared to healthy control cells. (e) Lipids that were specifically regulated in Hep3B + Wnt3a, HUH7 + Wnt3a, HUH7, SNU475 + Wnt3a and SNU475 as compared to healthy control cells.

Next, we compared the membrane lipidome profiles of untreated HCC cell lines to those of healthy cell lines, considering their direction of regulation. SNU475 and SNU398 cells showed the highest number of DRLs when compared to healthy control (Fig. S4B). In these cells, 20 DRLs were concordantly Up and 27 DRLs were concordantly Down. While many DRLs (28 Up and 31 Down in SNU475, 33 Up and 23 Down in SNU398) were specific to the two SNU cell lines, we also detected 48 DRLs (28 Up, 20 Down) that were shared in all five HCC cell lines.

To assess the effect of Wnt pathway manipulation by Wnt3a and Dkk1 treatment on DRLs detected in the membrane of HCC cells, we plotted a heatmap of lipid species and differential regulation contrasts in the HCC cells treated with Wnt3a or Dkk1 (Fig. S4C,D, Table S1). When compared to the healthy control cells, we termed the lipids that were Up or Down in untreated HCC cells as the “cancer effect.” If the regulation direction of such a lipid was reversed after treatment with Wnt3a or Dkk1 (i.e., from Up to Down or from Down to Up), we termed this influence as “treatment moderates cancer effect.” If the regulation direction of such a lipid was retained after treatment with Wnt3a or Dkk1 (i.e., Up & Up or Down & Down), we termed this influence as “treatment amplifies cancer effect.” There was also a group of lipids termed “treatment does not have effect” and not affected by Wnt pathway manipulation. The last group of lipids termed “side effect of treatment” were not affected in cancer but were Up or Down after Wnt pathway manipulation. We found that the membrane lipids of SNU475 and HUH7 cells were largely irresponsive to Wnt3a treatment (Fig. S4C, 266 lipids for SNU475 cells and 174 lipids for HUH7 cells represented in yellow bars). In SNU475 cells, we observed that Wnt3a treatment amplifies the cancer effect (42 lipids represented in red bars) rather than moderating it (33 lipids represented in green bars, Table S2). However, in HUH7 cells, Wnt3a treatment reduced the cancer effect (54 lipids represented in green bars, Table S2) rather than promoting it (22 lipids represented in red bars). In contrast to the SNU475 and HUH7 cells, Wnt3a treatment mostly mitigated the cancer effect by reversing the differential regulation of lipids in Hep3B cells (Fig. S4C, 113 lipids represented in green bars, Table S2). We also noticed that the number of lipids influenced by side effects of treatment (53 lipids represented in orange bars) or responded to treatment by amplifying the cancer effect (44 lipids represented in red bars) was higher in Hep3B cells than in SNU475 and HUH7 cells.

The response of SNU398 cells to Dkk1 treatment was very similar to that of SNU475 cells to Wnt3a treatment in that their membrane lipids were mostly irresponsive to treatment (Fig. S4D, 259 lipids represented in yellow bars). Apart from that, the moderating, amplifying, and side effects of Dkk1 treatment on cancer effect were comparable in SNU398 cells (48, 39, and 25 lipids represented in green, red, and orange bars, respectively). Interestingly, HepG2 cells were more responsive to Dkk1 treatment than SNU398 cells were, with less irresponsive lipids and more reversed/enhanced lipids (83, 44, and 63 lipids represented in yellow, green, and red bars, respectively). Taken together, these results indicate that the membrane lipids are differentially regulated between HCC cells and healthy cells and that certain DRLs further respond to Wnt pathway manipulation by either alleviating or enhancing the cancer effect.

### 3.5 DAG and ceramide regulate Wnt/β‐catenin signaling activity in SNU475 and HepG2 cells

Although the plasma membrane lipidome profiles of the HCC cells are in general significantly different from each other, certain lipids appear to be similarly regulated in response to manipulation of canonical Wnt signaling manipulation, most likely due to their conserved role in the regulation of signaling activity at the plasma membrane. Specifically, we found that the levels of DAG and ceramide at the plasma membrane decreased after activation of Wnt/β‐catenin signaling with Wnt3a treatment (Table S1). Both DAG and ceramide have been associated with Wnt signaling in developmental, homeostatic, and tumorigenic processes [19, 45, 46, 47]. The binding of the canonical Wnt ligand to the Fz receptor at the plasma membrane causes endocytic internalization of the Wnt‐receptor complex and co‐internalization of the membrane lipids that are found in the vicinity of the Wnt‐receptor complex [17]. Thus, the decline in the membrane levels of DAG and ceramide following Wnt3a treatment of HCC cells suggests that these lipids are involved in the formation of the Wnt‐receptor complex and downstream signaling activity.

To test this hypothesis, we selected two types of HCC cells for the mechanistic studies: (a) SNU475 cells that have a relatively lower level of endogenous Wnt/β‐catenin activity and can be stimulated with Wnt3a, (b) HepG2 cells that have a high level of endogenous Wnt/β‐catenin activity and cannot be stimulated with Wnt3a (Fig. 1A and Fig. S1A). On the one side, we supplied the cells with DAG or ceramide. On the other side, to deplete DAG and ceramide, we treated the cells with the enzyme DAG kinase (DGK), which phosphorylates and inactivates DAG, and the inhibitor of ceramide synthesis myriocin, respectively.

**Figure 2:**
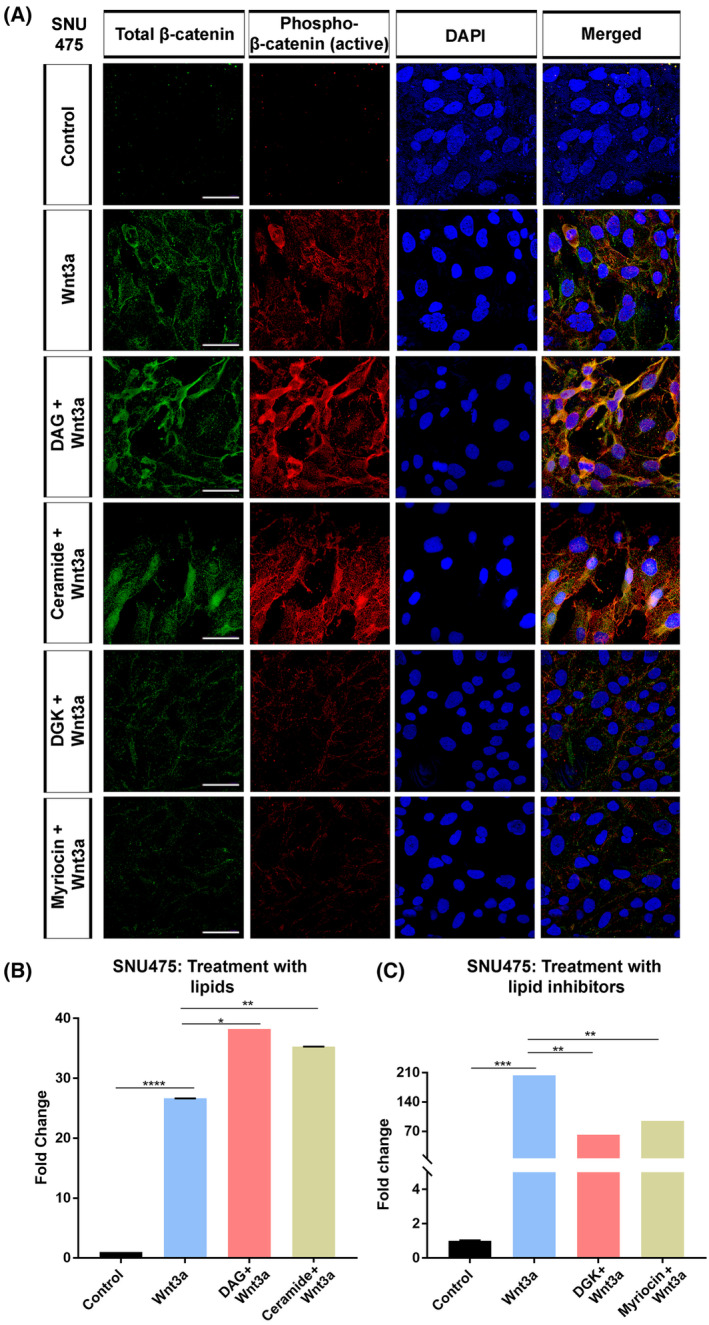
DAG and ceramide regulate Wnt/β‐catenin signaling activity in SNU475 cells. (A) Anti total β‐catenin (green) and anti‐phospho‐β‐catenin (red) staining of SNU475 cells. Cells are counterstained for DAPI. When compared to Wnt3a‐treated cells, treatment of cells with DAG or ceramide increases the expression of total and active β‐catenin while depletion of DAG with DGK or ceramide with myriocin decreases both types of β‐catenin. (B, C) Wnt/β‐catenin signaling activity (normalized to renilla luciferase activity) in Wnt3a‐treated SNU475 cells treated with (B) DAG or ceramide and (C) DGK or myriocin. Average and standard deviation of the mean (error bars) values of pBAR luciferase reporter activity are shown. Statistical significance was evaluated using an unpaired *t*‐test. ****P < 0.0001, ***P < 0.001, **P < 0.01, and *P < 0.05. Error bars represent standard deviation. Scale bars: 50 μm. Three independent experiments were performed.

SNU475 cells exhibited a dramatic increase in both total and phospho‐β‐catenin (active form, phosphorylated at Ser675 leading to nuclear localization and transcriptional activation) in groups first treated with DAG or ceramide and next treated with Wnt3a as compared to the control and Wnt3a‐treated groups (Fig. 2A). In contrast, depletion of the lipids with DGK or myriocin followed by Wnt3a treatment led to a decrease in β‐catenin levels. DAG and ceramide significantly increased the activation of the pBAR reporter of Tcf/Lef‐mediated transcription in SNU475 cells that were induced with Wnt3a (Fig. 2B). Both DGK and myriocin treatment strongly suppressed Wnt3a‐induced signaling activity in SNU475 cells (Fig. 2C). DAG addition to DGK‐treated SNU475 cells efficiently reversed the inhibitory effect of DGKA (Fig. S5A). To test the modulatory influence of DGK on the membrane DAG, we measured the DAG content in HepG2 cells and detected a significant reduction in DAG levels in response to DGK treatment (Fig. S6A). Immunofluorescence staining using a biotin‐conjugated DAG antibody further confirmed that Wnt3a stimulation increased the DAG levels, which were reduced by DGK treatment (Fig. S6B). DAG addition abolished the suppressor effect of DGK in Wnt3a‐induced cells. Transfection of HeLa cells with a plasmid containing DGKA caused a comparable reduction in β‐catenin levels after Wnt3a stimulation (Fig. S7A). DAG treatment in cells transfected with DGKA efficiently restored the β‐catenin levels back to levels detected after Wnt3a treatment. DGKA expression likewise suppressed activation of Tcf/Lef‐mediated transcription in HEK293T cells stimulated with either Wnt3a or LiCl (Fig. S7B).

**Figure 3:**
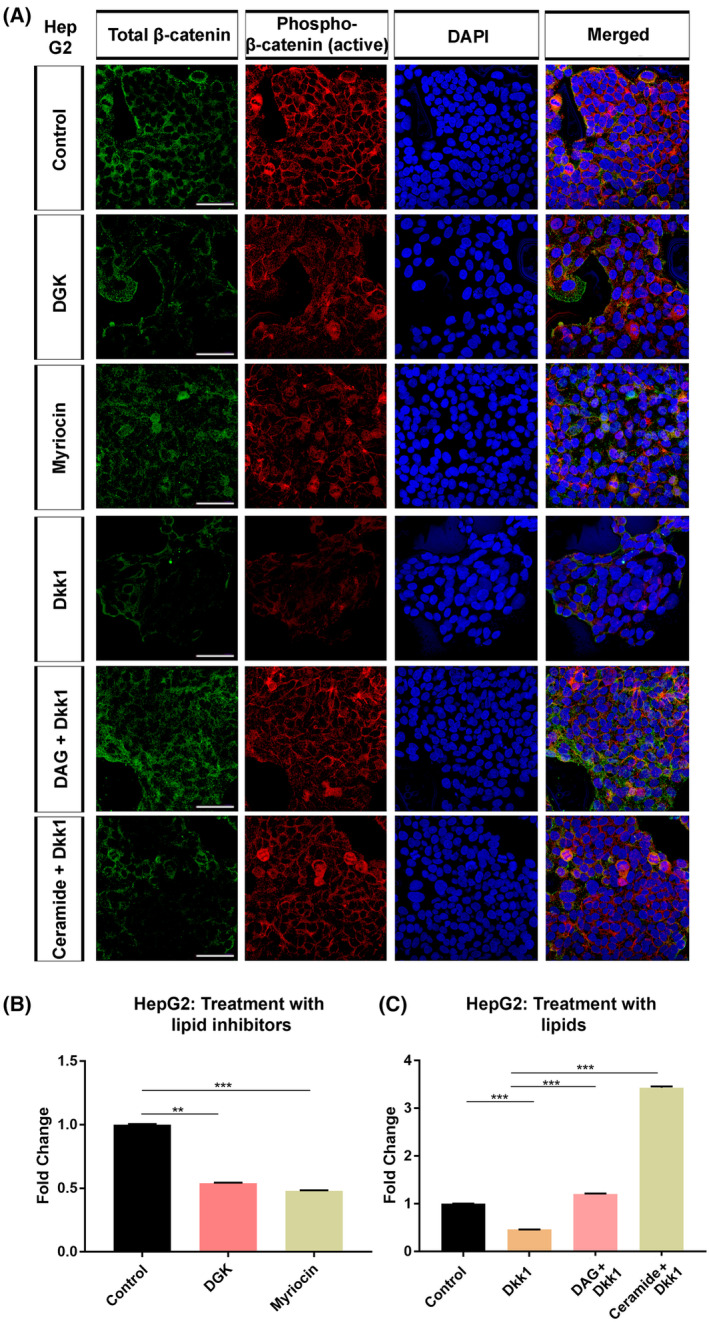
DAG and ceramide regulate Wnt/β‐catenin signaling activity in HepG2 cells. (A) Anti total β‐catenin (green) and anti‐phospho‐β‐catenin (red) staining of HepG2 cells. Cells are counterstained for DAPI. Depletion of DAG with DGK or ceramide with myriocin decreases the expression of total and active β‐catenin. Treatment with DAG or ceramide efficiently restores the expression of total and active β‐catenin, which are potently suppressed by the Wnt inhibitor dickkopf1 (Dkk1). (B, C) Wnt/β‐catenin signaling activity (normalized to renilla luciferase activity) in (B) HepG2 cells treated with DGK or myriocin and (C) Dkk1‐treated HepG2 cells treated with DAG or ceramide. Average and standard deviation of the mean (error bars) values of pBAR luciferase reporter activity are shown. Statistical significance was evaluated using an unpaired *t*‐test. ***P < 0.001 and **P < 0.01. Error bars represent standard deviation. Scale bars: 50 μm. Three independent experiments were performed.

In HepG2 cells, depletion of DAG or ceramide reduced endogenous total and active β‐catenin (Fig. 3A). On the other hand, DAG or ceramide restored the levels of both total and active β‐catenin, which were remarkably reduced in response to treatment with the Wnt inhibitor Dkk1, back to the control level. DGK and myriocin reduced endogenous canonical Wnt signaling activity in HepG2 cells, at least by 50% (Fig. 3B), while DAG rescued the signaling activity that was inhibited by Dkk1 and ceramide treatment resulted in a much stronger activation of Wnt signaling (Fig. 3C). DAG addition to DGK‐treated HepG2 cells recovered Wnt signaling activity and restored it to the control levels (Fig. S5B). Thus, DAG and ceramide are capable of regulating Wnt/β‐catenin signaling in HCC cells independently of whether their endogenous signaling activity is low or high.

### 3.6 DAG and ceramide induce caveolae‐mediated internalization of Wnt‐receptor complex in HCC cells

**Figure 4:**
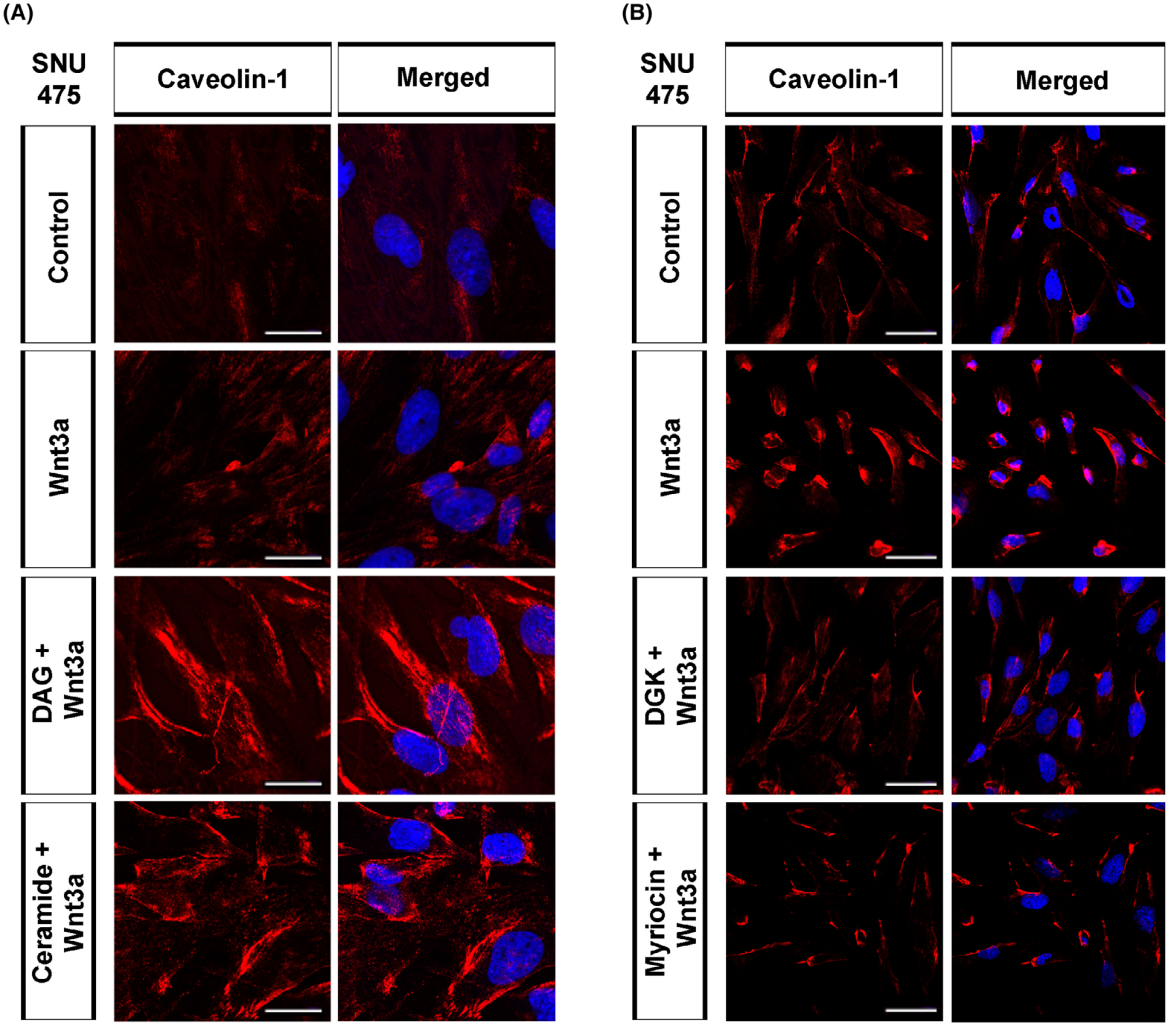
DAG and ceramide induce caveolae‐mediated internalization of Wnt‐receptor complex in HCC cells. Anti Caveolin‐1 (red) staining of SNU475 cells treated with (A) DAG or ceramide and (B) DGK or myriocin, and stimulated with Wnt3a CM. Cells are counterstained for DAPI. When compared with Wnt3a‐treated SNU475 cells, treatment of cells with DAG or ceramide strongly increases the expression of Caveolin‐1. Caveolin‐1 level, which increases in response to Wnt3a‐stimulation, is restored to control levels upon depletion of DAG with DGK or ceramide with myriocin. Scale bars: 25 μm (A) and 50 μm (B). Three independent experiments were performed.

Canonical Wnt‐receptor complex is internalized via the caveolae‐mediated endocytic pathway, leading to the activation of Wnt/β‐catenin signaling [19, 44]. To test whether the Wnt3a‐induced decrease in membrane DAG and ceramide levels is associated with caveolae‐mediated endocytosis, we examined caveolin‐1 levels in HCC cells treated with either lipids or lipid inhibitors. Immunofluorescence staining in SNU475 cells showed that of DAG and ceramide strongly enhanced the inductive effect of Wnt3a on caveolin‐1‐dependent endocytosis (Fig. 4A). Western blotting confirmed that DAG and ceramide increased the levels of caveolin‐1 in Wnt3a‐treated SNU475 cells, in which canonical Wnt signaling was successfully activated, observed by an increase in phospho‐β‐catenin levels (Fig. S8A). In contrast, caveolin‐1 levels decreased in Wnt3a‐treated SNU475 cells after DGK or myriocin treatment (Fig. 4B). Caveolin‐1 levels also decreased in HepG2 cells after DGK or myriocin treatment (Fig. S8B). Despite the inhibition of canonical Wnt signaling by DGK or myriocin observed by a reduction in phospho‐β‐catenin, caveolin‐1 was not detectable by western blotting in HepG2 cells, most likely due to low levels of endogenous caveolin‐1 (Fig. S8C). These results suggest that DAG and ceramide induce caveolin‐mediated endocytosis of the canonical Wnt‐receptor complex, thereby enhancing Wnt/β‐catenin signaling in HCC cells.

### 3.7 Depletion of DAG and ceramide impairs the ability of HCC cells to proliferate and grow into tumors

**Figure 5:**
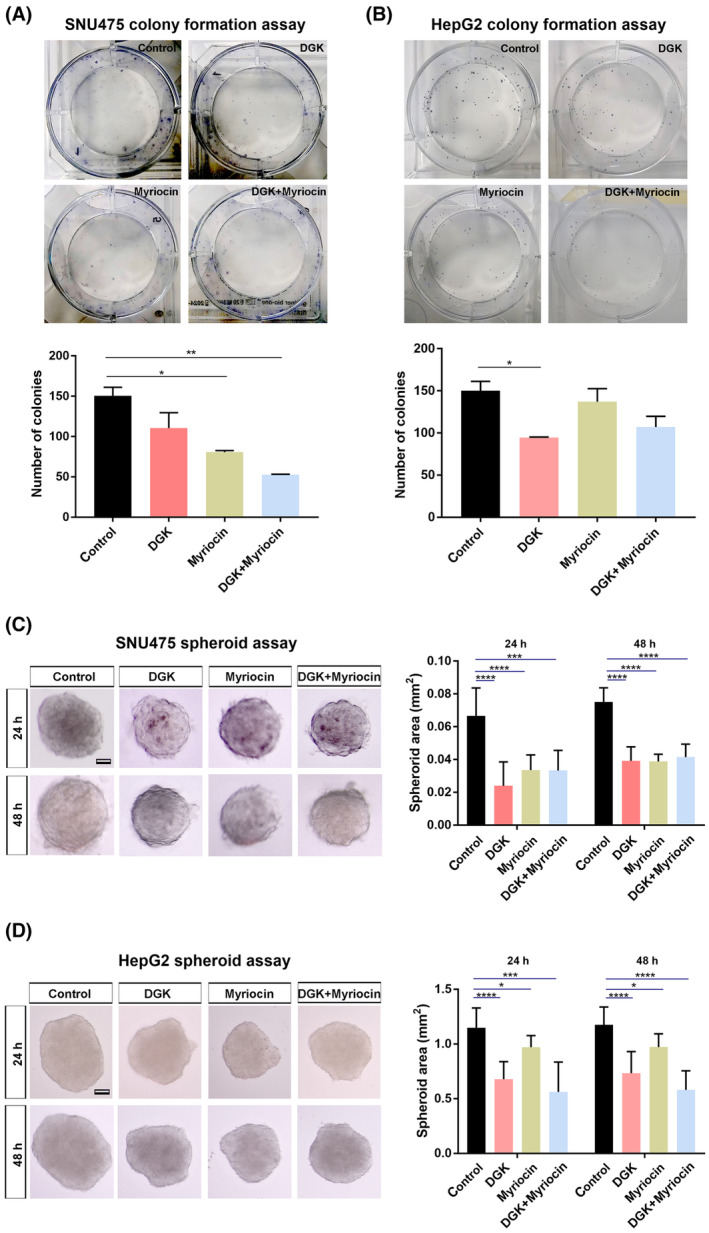
Depletion of DAG and ceramide impairs the ability of HCC cells to proliferate and grow into tumors. (A, B) Colony formation assay and quantification in (A) SNU475 cells and (B) HepG2 cells treated with DGK to deplete DAG, myriocin to deplete ceramide, or both. In SNU475 cells, the number of colonies significantly decreases after myriocin or a combination of inhibitors compared to the control. In HepG2 cells, the number of colonies significantly decreases after DGK treatment. (C, D) 3D spheroid formation assay and quantification of the spheroid area in (C) SNU475 cells and (D) HepG2 cells treated with DGK, myriocin, or both. In SNU475 cells, individual treatment of inhibitors reduces spheroid areas at both 24 and 48 h, while the combined treatment does not have an additive effect. In HepG2 cells, individual and combined treatment of inhibitors reduces spheroid areas at both 24 and 48 h. Statistical significance was evaluated using an unpaired *t*‐test. ****P < 0.0001, ***P < 0.001, **P < 0.01, and *P < 0.05. Error bars represent standard deviation. Scale bars: 500 μm. Three independent experiments were conducted.

To examine the effect of DAG and ceramide on HCC cell proliferation, we performed a colony formation assay on SNU475 and HepG2 cells. While depletion of DAG with DGK and ceramide with myriocin reduced the colony‐forming ability of SNU475 cells, a more prominent decrease was detectable when the cells were treated with both lipid synthesis inhibitors (Fig. 5A). We observed a similar reduction in the number of colonies formed by HepG2 cells treated with DGK, but not by those treated with myriocin (Fig. 5B). Yet, co‐treatment of HepG2 cells with DGK and myriocin showed a non‐significant reduction in the number of colonies.

Next, to test whether DAG and ceramide are involved in tumor growth, we exploited the spheroid formation assay, which is a scaffold‐free 3D model enabling quantitative analysis of tumor growth rate. Individual or combined treatment of SNU475 cells with the lipid synthesis inhibitors DGK and myriocin resulted in a significant reduction of the spheroid area at both 24 and 48 h after the addition of the inhibitors (Fig. 5C). DGK and myriocin likewise efficiently suppressed the growth of HepG2 spheroids (Fig. 5D). These results collectively suggest that the reduction of DAG and ceramide efficiently suppresses the proliferation and growth of HCC cells.

### 3.8 Depletion of DAG and ceramide reduces the migration capacity of HCC cells *in vivo*

**Figure 6:**
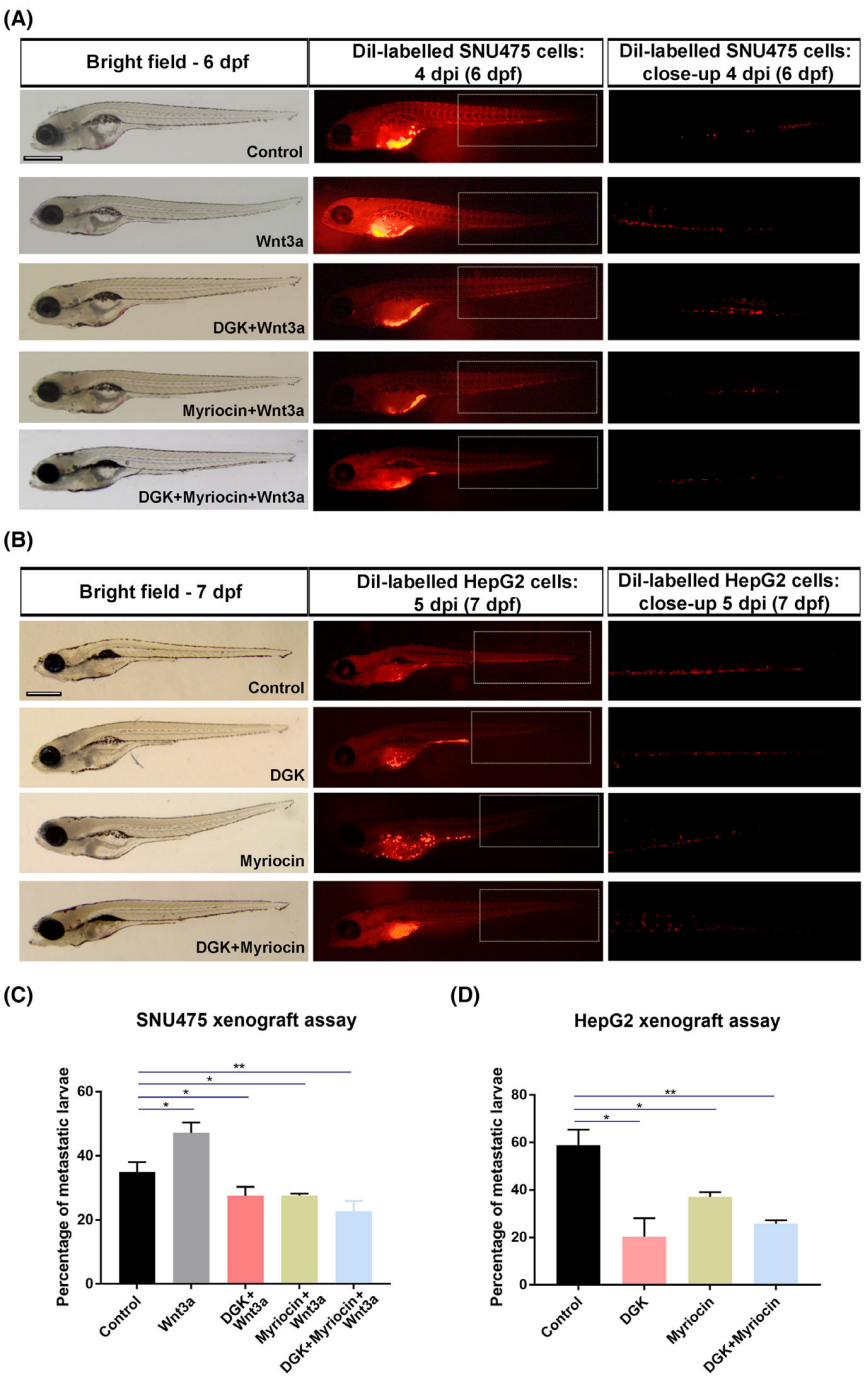
Depletion of DAG and ceramide reduces the migration capacity of HCC cells *in vivo*. (A) Representative bright‐field and fluorescence microscope images of 6 dpf zebrafish embryos injected with SNU475 cells pre‐treated with Wnt3a, DGK + Wnt3a, myriocin+Wnt3a or DGK + myriocin+Wnt3a into the yolk sac of 2 dpf zebrafish larvae. While Wnt3a increases the migration capacity of SNU475 cells, individual and combined treatment of inhibitors strongly reduce this capacity even below the control levels. (B) Representative bright‐field and fluorescence microscope images of 7 dpf zebrafish embryos injected with HepG2 cells pre‐treated with DGK, myriocin, or DGK + myriocin into the yolk sac of 2 dpf zebrafish larvae. Individual and combined treatment of inhibitors dramatically reduces the migration capacity of HepG2 cells. (C, D) Quantification of metastatic larvae injected with (C) SNU475 and (D) HepG2 cells. Statistical significance was evaluated using Student's *t*‐test. **P < 0.01, and *P < 0.05. Error bars represent standard deviation. Scale bars: 50 μm. Three independent experiments were conducted.

Aberrant regulation of cell migration drives metastasis. To examine the effect of DAG and ceramide on HCC cell migration and metastasis, we exploited the zebrafish model. We generated larval xenografts by injecting DiI‐labeled SNU475 or HepG2 cells into the yolk sac at 2 dpf, treated the xenografts with lipid synthesis inhibitors, and monitored the cancer cell behavior over time. At 4 days post‐injection (dpi), activation of canonical Wnt signaling with Wnt3a resulted in increased migration of SNU475 cells as compared to the control (Fig. 6A). Individual or combined depletion of DAG with DGK and ceramide with myriocin in Wnt3a‐treated SNU475 cells remarkably reduced their migration ability. DGK and myriocin likewise reduced the migration capacity of HepG2 cells that almost completely invaded the caudal tissue and formed micrometastasis colonies (Fig. 6B). The inhibitory effect of DAG or ceramide depletion on HCC cell migration was also reflected in the number of metastatic larvae transplanted with SNU475 cells (Fig. 6C) or HepG2 cells (Fig. 6D). Thus, the depletion of DAG and ceramide can suppress the migratory capacity of HCC cells.

## 4 Discussion

**Figure 7:**
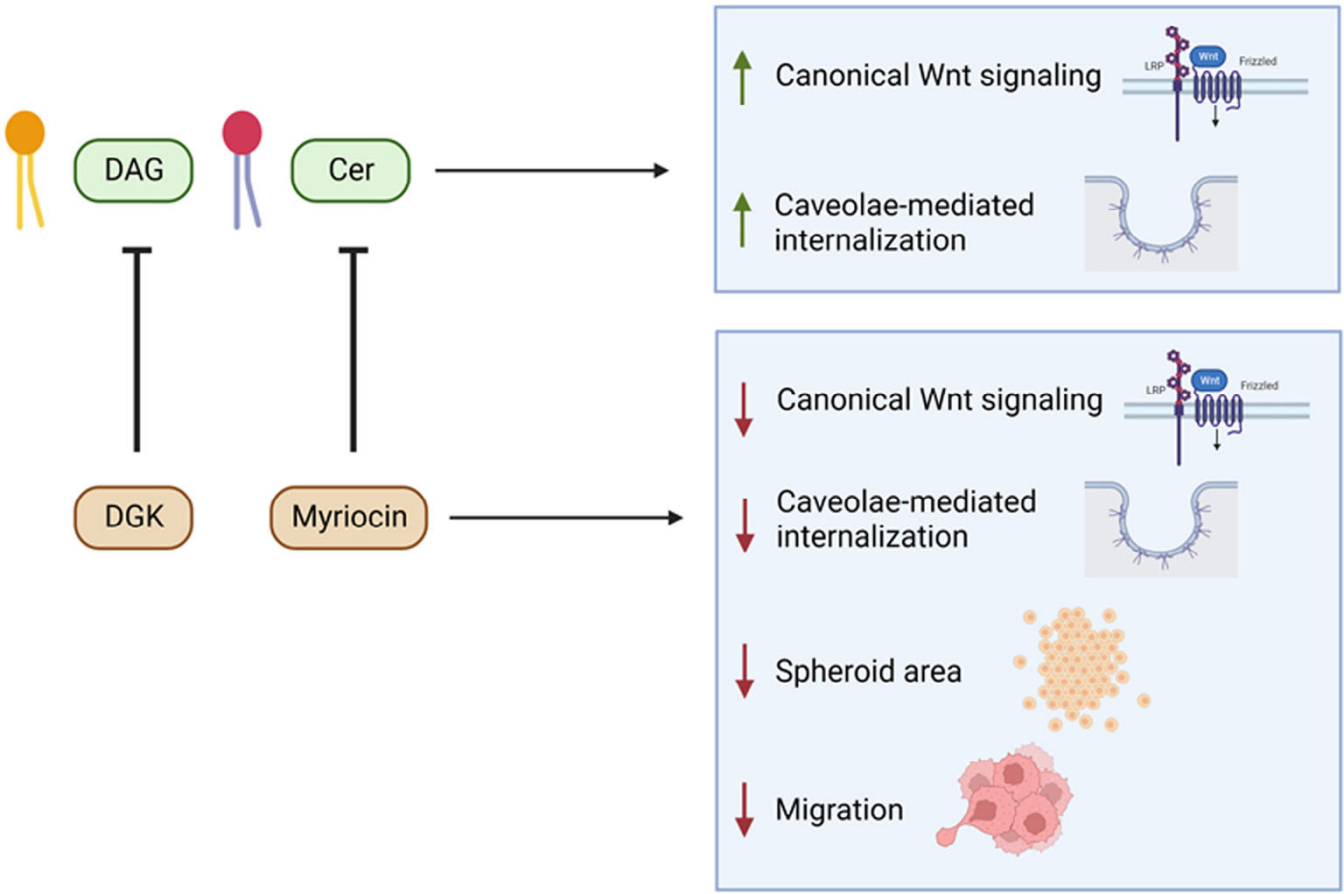
DAG and ceramide act as key regulators of Wnt/β‐catenin signaling and tumor growth. DAG and ceramide promote canonical Wnt signaling activity and caveolae‐mediated internalization of the Wnt‐receptor complex in HCC cells. On the other hand, inhibition of these lipids with DGK or myriocin, respectively, reduces canonical Wnt signaling activity, caveolae‐mediated internalization, spheroid area, and cell migration.

A large‐scale study of the lipidome, that is, the complete lipid profile of biological systems, at the quantitative level by mass spectrometry‐based lipidomics provides a reasonable approximation of the plasma membrane environment by uncovering membrane lipid composition [35, 48, 49]. Thus, lipidome profiling is a valuable tool to identify the roles of membrane lipids in cellular processes as well as to reveal how lipid composition is altered in cancer. Nevertheless, there is limited information on the lipidome profiles of cancer cells and no data on the comparative analysis of their plasma membrane lipidome profiles. To our knowledge, this is the first study to compare the lipidome profiles of the plasma membranes in different HCC cells. Moreover, we provide the first evidence that manipulation of Wnt/β‐catenin signaling dramatically changes the membrane lipid composition in HCC cells. Accordingly, we have reached the following conclusions: (a) Six different HCC cells, that is, HUH7, SNU475, Hep3B, HepG2, SNU398, and Mahlavu cells, vary in their level of endogenous Wnt/β‐catenin signaling activity and significantly differ concerning their plasma membrane lipid composition. (b) HCC cells alter their plasma membrane lipid composition in response to Wnt pathway activation and inhibition. (c) The membrane lipidome profiles of HCC cells differ dramatically from those of healthy cells. There is a considerable amount of lipids that are both differentially regulated in HCC cells and responsive to canonical Wnt pathway manipulation in these cells. (d) DAG and ceramide, which are downregulated after Wnt3a treatment in HCC cells' plasma membrane, enhance Wnt/β‐catenin signaling activity in SNU475 and HepG2 cells. In contrast, their depletion in cells suppresses Wnt signaling activity. (e) DAG and ceramide can enhance caveolin‐mediated endocytosis of the canonical Wnt‐receptor complex. (f) Depletion of DAG and ceramide and interferes with the proliferation, tumor formation, and *in vivo* migration capacity of SNU475 and HepG2 cells (Fig. 7).

By exhibiting aberrant activation of Wnt/β‐catenin signaling, HCC would thus constitute a great platform for comparative lipidomics analysis of the plasma membrane [50, 51, 52]. Comprehensive analysis of the membrane lipidome profiles of HCC cells and healthy control liver cells revealed that while the number of sterols and sphingolipids were generally lower in the HCC cells than in the healthy cells, glycerophospholipids, and their intermediates were more abundant in the HCC cells. Specifically, CE, Cer, HexCer, and SM (except Mahlavu) decreased, whereas LPA, LPC, LPE, LPI, LPS, PI, and PS increased significantly in HCC cell lines compared to the healthy cells. However, we were not able to detect a particular lipidome profile that distinguishes the HCC cells with high endogenous canonical Wnt signaling activity, that is, HepG2 and SNU398 cells, albeit the overall number of glycerophospholipids were lower in HepG2 cells. Therefore, we decided to examine the response of HCC cells to Wnt signaling manipulation concerning the alteration of the membrane lipids. In general, all lipid categories except for the glycerophospholipids and lipid classes except for PC and PE were downregulated in HUH7, SNU475, and Hep3B cells where canonical Wnt signaling was activated with Wnt3a treatment. This downregulation influence was most prominent in the levels of Cer, DAG, and SM that have previously been associated with the specialized ordered membrane domains, the so‐called lipid rafts [53, 54]. Ordered membrane domains are dynamically assembled from various saturated lipids, sterols, sphingolipids, and lipid‐anchored proteins [55]. Canonical Wnt ligands, including Wnt3a, bind to their receptors within the ordered domains [17]. Canonical Wnt‐receptor complex induces recruitment of the cytoplasmic effector Disheveled to the membrane and formation of the Wnt signalosome [56]. A key step in Wnt pathway regulation is the internalization of the Wnt signalosome, a critical balance that ensures both pathway activation and endocytic degradation of excessive ligands and receptors [19]. Thus, Wnt signalosome internalization inextricably brings about co‐internalization of the particular ordered domain lipids that are packed around the Wnt‐receptor complex, resulting in their downregulation at the membrane. This outcome is in line with our previous findings where canonical Wnt treatment reduced the generalized polarization of the membrane, indicative of reduced membrane order [17].

Wnt3a has been shown to induce caveolin‐dependent (clathrin‐independent) internalization of the Wnt‐receptor complex to activate canonical Wnt signaling, while Dkk1 is associated with clathrin‐dependent internalization of Lrp6 followed by pathway inhibition [44]. In addition, canonical Wnt ligands prefer to bind their receptor complex in the caveolin‐rich ordered membrane domains, that is, the lipid rafts, in contrast to Dkk1 reducing the distribution of Lrp6 in these domains. Nevertheless, evidence from subsequent studies has reported a role for clathrin also on canonical Wnt signaling by enhancing Wnt signalosome formation and mediating endocytosis of the Wnt‐receptor complex [19, 56, 57, 58]. Since caveolin‐dependent and clathrin‐dependent endocytosis take place in the ordered domains and disordered domains, respectively, treatment with Wnt3a and Dkk1 are expected to alter the membrane lipid composition differently [59]. In HCC cells, when compared with the Wnt3a treatment, the Dkk1 treatment resulted in the downregulation of far more lipids in virtually all lipid categories and classes. Owing to its association with the ordered membrane domains, Wnt3a was rather influential on the ordered domains, a restricted portion of the membrane, most likely resulting in a balanced regulation of membrane lipids following internalization. In contrast, due to the expanded nature of clathrin‐dependent endocytosis throughout the membrane, Dkk1 treatment appeared to overwhelm a wider part of the membrane and caused the downregulation of a larger number of lipids. It is also noteworthy that treatment with Wnt3a or Dkk1 resulted in the downregulation of Cer in all HCC cells and DAG in all but HepG2 + Dkk1 cells. Our results have further revealed that ceramide and DAG enhanced caveolin‐mediated endocytosis of the canonical Wnt‐receptor complex to activate Wnt/β‐catenin signaling in SNU475 cells, while their depletion suppressed caveolin‐mediated endocytosis in HepG2 cells that have relatively higher level of Wnt signaling activity. These results suggest that Cer and DAG at the plasma membrane act as common players in the regulation of canonical Wnt signaling in response to Wnt3a‐mediated activation or Dkk1‐mediated inhibition of the pathway and that these lipids appear to directly influence caveolin‐mediated endocytosis of the Wnt‐receptor complex.

Comprehensive bioinformatic analyses of membrane lipidome profiles of the Wnt3a/Dkk1‐treated HCC cells, the untreated HCC cells, and the healthy control cells revealed a clear distinction in the clustering patterns of DRLs. Strikingly, this difference was most pronounced in the clustering of the DRLs detected in the comparison of the HCC cells, regardless of whether they were treated or not, to the healthy cells. The difference in DRLs between the treated and the untreated HCC cells was rather mild, leading to a weaker clustering. Thus, for the membrane lipid composition of an HCC cell, the influence of being cancerous is stronger than that of having abnormal Wnt signaling activity. There was a considerable number of lipids that were differentially regulated in all HCC cells. When examined further, some of these DRLs were oppositely regulated between cancer and pathway manipulation. In other words, in a particular HCC cell line, several lipids were upregulated as compared to the healthy control line and downregulated after manipulation of the Wnt pathway, and several lipids were downregulated compared to the control and upregulated after pathway manipulation. We termed this restoration effect of pathway manipulation as “moderation of cancer by Wnt pathway treatment” and marked the DRLs that mediate this effect in “green.” Overall, the “green” lipids that were oppositely responsive to cancer formation and Wnt pathway manipulation could be promising targets for the treatment of HCC.

Based on the membrane lipidomics analysis, we found that Cer and DAG decreased significantly in response to the activation of the canonical Wnt pathway in all three HCC cells treated with Wnt3a. Our mechanistic experiments in SNU475 cells (low level of endogenous Wnt/β‐catenin activity and can be stimulated with Wnt3a) and HepG2 cells (high level of endogenous Wnt/β‐catenin activity and cannot be stimulated with Wnt3a) revealed Cer and DAG were able to enhance Wnt signaling. On the other hand, the depletion of Cer and DAG potently inhibited Wnt signaling in these cells and efficiently suppressed their proliferation, tumoral growth, and migration capacity. Importantly, Cer and DAG were among the “green” lipids, strongly suggesting that they moderate the cancer effect. The neutral sphingomyelinase 2 enzyme has been shown to induce the production of Cer, a sphingolipid component of ordered membrane domains, which induces receptor clustering and plasma membrane curvature in the neural crest during development [54, 60]. Subsequent endocytosis of Wnt and BMP signaling complexes was sufficient to activate epithelial‐to‐mesenchymal transition, suggesting a key role for Cer in regulating cell migration. Thus, the decrease in Cer in Wnt3a‐treated HCC cells is likely due to its endocytosis with the Wnt‐receptor complex. In a different study, ceramides have been proposed to increase the level of exosomal miRNAs that activate Wnt/β‐catenin signaling and enhance cell invasion [61]. This is in line with the inhibition of cell migration upon depletion of Cer in SNU475 and HepG2 cells. Furthermore, ceramide metabolism has been reported to be dysregulated in HCC [62, 63, 64]. DAG is a key signaling molecule that has been identified as a component of the ordered membrane domains [65, 66, 67]. Elevated levels of DAG promote colony‐stimulating factor 1‐dependent proliferation in DGK knock‐out mice and modulate β‐catenin/cyclinD1 levels in osteoclast precursors [68]. DAG has also been associated with HCC progression [69, 70]. DAG, by either DGK‐mediated phosphorylation into phosphatidic acid or activating protein kinase C, supports tumor growth, and HCC progression [70, 71, 72]. We observed a parallel response to DAG in SNU475 and HepG2 cell lines. Therefore, Cer and DAG harbor a potential for diagnosis and targeted therapy in patients with HCC, especially when Wnt/β‐catenin signaling is aberrantly activated.

Liver lipidome has been examined in tissue and serum samples of patients with HCC or non‐alcoholic fatty liver disease (NAFLD), a chronic liver disease and the fastest‐growing cause of HCC [73, 74, 75, 76, 77]. Due to the liver's essential roles in lipid metabolism, altered lipid metabolism in hepatic cells contributes to the progression of HCC, by providing the neoplastic cells with energy and supporting the growth of HCC lesions in humans [78]. The changes in lipid metabolism and the levels of different lipids have been associated with the severity of HCC [75]. However, these studies are mainly based on the data obtained from whole tissues or serum, presumably leading to heterogeneity in the role of lipids in HCC. As our data reveal that various lipids, including Cer and DAG, are differentially regulated between different types of HCC, it is also very likely that their functions vary depending on the cancer subtype. Thus, membrane lipidome analysis is a key aspect for individual and cell type‐dependent identification and characterization of the lipids in cancer.

## 5 Conclusion

Lipids are essential components of the plasma membrane that is known to alter significantly in cancer cells as compared to normal cells. Comparative lipidome profiling of the plasma membranes of cancer cells and healthy cells can greatly contribute to our understanding of the role of specific membrane lipids in the onset and progression of cancer and to the identification of specific lipids including Cer and DAG that could serve as effective diagnostic or prognostic biomarkers and therapeutic targets in cancer.

## Conflict of interest

The authors declare no conflict of interest.

## Author contributions

GO and YA designed the experiments. YA, MK, and DI performed molecular and cell biology experiments. YD and GH conducted the bioinformatics analyses. YA drafted the manuscript, and YD, GH, and MK contributed to the results, materials, and methods. GO wrote the manuscript.

### Peer review

The peer review history for this article is available at https://www.webofscience.com/api/gateway/wos/peer‐review/10.1002/1878‐0261.13520.

## Supporting information


**Fig. S1.** Different HCC cell lines vary in their activity of Wnt/β‐catenin signaling and plasma membrane lipid composition.
**Fig. S2.** Plasma membranes of different HCC cells diverge with respect to distribution of main lipid categories and lipid species.
**Fig. S3.** Membrane lipids of HCC cells alter significantly in response to inhibition of Wnt/β‐catenin signaling.
**Fig. S4.** Global comparison of membrane lipidome profiles reveals differential regulation of lipids in HCC cells and healthy cells.
**Fig. S5.** DAG and ceramide restore Wnt/β‐catenin signaling activity after DGK treatment in SNU475 and HepG2 cells.
**Fig. S6.** DGK reduces membrane DAG in HepG2 and HEK293T cells.
**Fig. S7.** DGKA transfection reduces Wnt/β‐catenin signaling activity in HeLa and HEK293T cells.
**Fig. S8.** Depletion of DAG or ceramide reduces caveolae‐mediated internalization of Wnt‐receptor complex in HepG2 cells.

**Table S1.** Differentially regulated lipids at the plasma membrane of various HCC cells.

**Table S2.** Lipids moderated by treatment (Wnt3a/Dkk1).

## Data Availability

The data that supports the findings of this study are available in figures, tables, and the supplementary material of this article.

## References

[1] Wang W , Smits R , Hao H , He C . Wnt/β‐catenin signaling in liver cancers. Cancers. 2019;11:926. 10.3390/cancers11070926 31269694 PMC6679127

[2] Duchartre Y , Kim YM , Kahn M . The Wnt signaling pathway in cancer. Crit Rev Oncol Hematol. 2016;99:141–149. 10.1016/j.critrevonc.2015.12.005 26775730 PMC5853106

[3] Nusse R , Varmus HE . Many tumors induced by the mouse mammary tumor virus contain a provirus integrated in the same region of the host genome. Cell. 1982;31:99–109. 10.1016/0092-8674(82)90409-3 6297757

[4] Tsukamoto AS , Grosschedl R , Guzman RC , Parslow T , Varmus HE . Expression of the int‐1 gene in transgenic mice is associated with mammary gland hyperplasia and adenocarcinomas in male and female mice. Cell. 1988;55:619–625. 10.1016/0092-8674(88)90220-6 3180222

[5] Khalaf AM , Fuentes D , Morshid AI , Burke MR , Kaseb AO , Hassan M , et al. Role of Wnt/β‐catenin signaling in hepatocellular carcinoma, pathogenesis, and clinical significance. J Hepatocell Carcinoma. 2018;5:61–73. 10.2147/jhc.s156701 29984212 PMC6027703

[6] Waisberg J , Saba GT . Wnt−/−β‐catenin pathway signaling in human hepatocellular carcinoma. World J Hepatol. 2015;7:2631–2635. 10.4254/wjh.v7.i26.2631 26609340 PMC4651907

[7] Caruso S , Calatayud AL , Pilet J , La Bella T , Rekik S , Imbeaud S , et al. Analysis of liver cancer cell lines identifies agents with likely efficacy against hepatocellular carcinoma and markers of response. Gastroenterology. 2019;157:760–776. 10.1053/j.gastro.2019.05.001 31063779

[8] Rebouissou S , Nault JC . Advances in molecular classification and precision oncology in hepatocellular carcinoma. J Hepatol. 2020;72:215–229. 10.1016/j.jhep.2019.08.017 31954487

[9] Karabicici M , Azbazdar Y , Ozhan G , Senturk S , Firtina Karagonlar Z , Erdal E . Changes in Wnt and TGF‐β signaling mediate the development of regorafenib resistance in hepatocellular carcinoma cell line HuH7. Front Cell Dev Biol. 2021;9:639779. 10.3389/fcell.2021.639779 34458250 PMC8386122

[10] Kwee SA , Tiirikainen M . Beta‐catenin activation and immunotherapy resistance in hepatocellular carcinoma: mechanisms and biomarkers. Hepatoma Res. 2021;7:8. 10.20517/2394-5079.2020.124 33553649 PMC7861492

[11] Ruiz de Galarreta M , Bresnahan E , Molina‐Sánchez P , Lindblad KE , Maier B , Sia D , et al. β‐Catenin activation promotes immune escape and resistance to anti‐PD‐1 therapy in hepatocellular carcinoma. Cancer Discov. 2019;9:1124–1141. 10.1158/2159-8290.cd-19-0074 31186238 PMC6677618

[12] Vilchez V , Turcios L , Marti F , Gedaly R . Targeting Wnt/β‐catenin pathway in hepatocellular carcinoma treatment. World J Gastroenterol. 2016;22:823–832. 10.3748/wjg.v22.i2.823 26811628 PMC4716080

[13] Demirci Y , Cucun G , Poyraz YK , Mohammed S , Heger G , Papatheodorou I , et al. Comparative transcriptome analysis of the regenerating zebrafish telencephalon unravels a resource with key pathways during two early stages and activation of Wnt/β‐catenin signaling at the early wound healing stage. Front Cell Dev Biol. 2020;8:584604. 10.3389/fcell.2020.584604 33163496 PMC7581945

[14] Ozhan G , Weidinger G . Restoring tissue homeostasis: Wnt signaling in tissue regeneration after acute injury. In: Hoppler SP , Moon RT , editors. Wnt signaling in development and disease: molecular mechanisms and biological functions. Hoboken: Wiley‐Blackwell; 2014.

[15] Steinhart Z , Angers S . Wnt signaling in development and tissue homeostasis. Development. 2018;145:dev146589. 10.1242/dev.146589 29884654

[16] Stamos JL , Weis WI . The β‐catenin destruction complex. Cold Spring Harb Perspect Biol. 2013;5:a007898. 10.1101/cshperspect.a007898 23169527 PMC3579403

[17] Sezgin E , Azbazdar Y , Ng XW , Teh C , Simons K , Weidinger G , et al. Binding of canonical Wnt ligands to their receptor complexes occurs in ordered plasma membrane environments. FEBS J. 2017;284:2513–2526. 10.1111/febs.14139 28626941 PMC5599997

[18] MacDonald BT , Tamai K , He X . Wnt/beta‐catenin signaling: components, mechanisms, and diseases. Dev Cell. 2009;17:9–26. 10.1016/j.devcel.2009.06.016 19619488 PMC2861485

[19] Azbazdar Y , Karabicici M , Erdal E , Ozhan G . Regulation of Wnt signaling pathways at the plasma membrane and their misregulation in cancer. Front Cell Dev Biol. 2021;9:631623. 10.3389/fcell.2021.631623 33585487 PMC7873896

[20] Jeong W , Jho EH . Regulation of the low‐density lipoprotein receptor‐related protein LRP6 and its association with disease: Wnt/β‐catenin signaling and beyond. Front Cell Dev Biol. 2021;9:714330. 10.3389/fcell.2021.714330 34589484 PMC8473786

[21] Karabicici M , Azbazdar Y , Iscan E , Ozhan G . Misregulation of Wnt signaling pathways at the plasma membrane in brain and metabolic diseases. Membranes. 2021;11. 10.3390/membranes11110844 PMC8621778 34832073

[22] Ozhan G . Editorial: Wnt signaling at the plasma membrane: activation, regulation and disease connection. Front Cell Dev Biol. 2021;9:780163. 10.3389/fcell.2021.780163 34760896 PMC8573280

[23] Jacobson K , Liu P , Lagerholm BC . The lateral organization and mobility of plasma membrane components. Cell. 2019;177:806–819. 10.1016/j.cell.2019.04.018 31051105 PMC6541401

[24] Sarmento MJ , Hof M , Šachl R . Interleaflet coupling of lipid nanodomains – insights from in vitro systems. Front Cell Dev Biol. 2020;8:284. 10.3389/fcell.2020.00284 32411705 PMC7198703

[25] Choromańska A , Chwiłkowska A , Kulbacka J , Baczyńska D , Rembiałkowska N , Szewczyk A , et al. Modifications of plasma membrane organization in cancer cells for targeted therapy. Molecules. 2021;26:1850. 10.3390/molecules26071850 33806009 PMC8037978

[26] Perrotti F , Rosa C , Cicalini I , Sacchetta P , Del Boccio P , Genovesi D , et al. Advances in lipidomics for cancer biomarkers discovery. Int J Mol Sci. 2016;17:1992. 10.3390/ijms17121992 27916803 PMC5187792

[27] Pradas I , Huynh K , Cabré R , Ayala V , Meikle PJ , Jové M , et al. Lipidomics reveals a tissue‐specific fingerprint. Front Physiol. 2018;9:1165. 10.3389/fphys.2018.01165 30210358 PMC6121266

[28] Bernardes N , Fialho AM . Perturbing the dynamics and organization of cell membrane components: a new paradigm for cancer‐targeted therapies. Int J Mol Sci. 2018;19. 10.3390/ijms19123871 PMC6321595 30518103

[29] Liu Z , Zhang Z , Mei H , Mao J , Zhou X . Distribution and clinical relevance of phospholipids in hepatocellular carcinoma. Hepatol Int. 2020;14:544–555. 10.1007/s12072-020-10056-8 32504407 PMC7366576

[30] Pakiet A , Kobiela J , Stepnowski P , Sledzinski T , Mika A . Changes in lipids composition and metabolism in colorectal cancer: a review. Lipids Health Dis. 2019;18:29. 10.1186/s12944-019-0977-8 30684960 PMC6347819

[31] Szlasa W , Zendran I , Zalesińska A , Tarek M , Kulbacka J . Lipid composition of the cancer cell membrane. J Bioenerg Biomembr. 2020;52:321–342. 10.1007/s10863-020-09846-4 32715369 PMC7520422

[32] Nelson CD , Perry SJ , Regier DS , Prescott SM , Topham MK , Lefkowitz RJ . Targeting of diacylglycerol degradation to M1 muscarinic receptors by beta‐arrestins. Science. 2007;315:663–666. 10.1126/science.1134562 17272726

[33] Özhan G , Sezgin E , Wehner D , Pfister AS , Kühl SJ , Kagermeier‐Schenk B , et al. Lypd6 enhances Wnt/β‐catenin signaling by promoting Lrp6 phosphorylation in raft plasma membrane domains. Dev Cell. 2013;26:331–345. 10.1016/j.devcel.2013.07.020 23987510

[34] Sezgin E , Kaiser HJ , Baumgart T , Schwille P , Simons K , Levental I . Elucidating membrane structure and protein behavior using giant plasma membrane vesicles. Nat Protoc. 2012;7:1042–1051. 10.1038/nprot.2012.059 22555243

[35] Sampaio JL , Gerl MJ , Klose C , Ejsing CS , Beug H , Simons K , et al. Membrane lipidome of an epithelial cell line. Proc Natl Acad Sci U S A. 2011;108:1903–1907. 10.1073/pnas.1019267108 21245337 PMC3033259

[36] Ejsing CS , Sampaio JL , Surendranath V , Duchoslav E , Ekroos K , Klemm RW , et al. Global analysis of the yeast lipidome by quantitative shotgun mass spectrometry. Proc Natl Acad Sci U S A. 2009;106:2136–2141. 10.1073/pnas.0811700106 19174513 PMC2650121

[37] Surma MA , Herzog R , Vasilj A , Klose C , Christinat N , Morin‐Rivron D , et al. An automated shotgun lipidomics platform for high throughput, comprehensive, and quantitative analysis of blood plasma intact lipids. Eur J Lipid Sci Technol. 2015;117:1540–1549. 10.1002/ejlt.201500145 26494980 PMC4606567

[38] Herzog R , Schuhmann K , Schwudke D , Sampaio JL , Bornstein SR , Schroeder M , et al. LipidXplorer: a software for consensual cross‐platform lipidomics. PLoS One. 2012;7:e29851. 10.1371/journal.pone.0029851 22272252 PMC3260173

[39] Herzog R , Schwudke D , Schuhmann K , Sampaio JL , Bornstein SR , Schroeder M , et al. A novel informatics concept for high‐throughput shotgun lipidomics based on the molecular fragmentation query language. Genome Biol. 2011;12:R8. 10.1186/gb-2011-12-1-r8 21247462 PMC3091306

[40] Conway J . UpSetR: a more scalable alternative to Venn and Euler diagrams for visualizing intersecting sets. *R package version* 140. 2017.

[41] Gu Z , Eils R , Schlesner M . Complex heatmaps reveal patterns and correlations in multidimensional genomic data. Bioinformatics. 2016;32:2847–2849. 10.1093/bioinformatics/btw313 27207943

[42] Kolde R . Pheatmap: pretty heatmaps. *R package version* 1012. 2019.

[43] Wickham H . Ggplot2: elegant graphics for data analysis. New York: Springer‐Verlag; 2016.

[44] Yamamoto H , Sakane H , Yamamoto H , Michiue T , Kikuchi A . Wnt3a and Dkk1 regulate distinct internalization pathways of LRP6 to tune the activation of beta‐catenin signaling. Dev Cell. 2008;15:37–48. 10.1016/j.devcel.2008.04.015 18606139

[45] Fu L , Deng R , Huang Y , Yang X , Jiang N , Zhou J , et al. DGKA interacts with SRC/FAK to promote the metastasis of non‐small cell lung cancer. Cancer Lett. 2022;532:215585. 10.1016/j.canlet.2022.215585 35131384

[46] Oh JH , Jeong KH , Kim JE , Kang H . Synthesized ceramide induces growth of dermal papilla cells with potential contribution to hair growth. Ann Dermatol. 2019;31:164–174. 10.5021/ad.2019.31.2.164 33911565 PMC7992683

[47] Pepperl J , Reim G , Lüthi U , Kaech A , Hausmann G , Basler K . Sphingolipid depletion impairs endocytic traffic and inhibits Wingless signaling. Mech Dev. 2013;130:493–505. 10.1016/j.mod.2013.04.001 23665457

[48] Butler LM , Mah CY , Machiels J , Vincent AD , Irani S , Mutuku SM , et al. Lipidomic profiling of clinical prostate cancer reveals targetable alterations in membrane lipid composition. Cancer Res. 2021;81:4981–4993. 10.1158/0008-5472.can-20-3863 34362796

[49] Klose C , Surma MA , Gerl MJ , Meyenhofer F , Shevchenko A , Simons K . Flexibility of a eukaryotic lipidome – insights from yeast lipidomics. PLoS One. 2012;7:e35063. 10.1371/journal.pone.0035063 22529973 PMC3329542

[50] Liu LJ , Xie SX , Chen YT , Xue JL , Zhang CJ , Zhu F . Aberrant regulation of Wnt signaling in hepatocellular carcinoma. World J Gastroenterol. 2016;22:7486–7499. 10.3748/wjg.v22.i33.7486 27672271 PMC5011664

[51] Liu Y , Zhou R , Yuan X , Han N , Zhou S , Xu H , et al. DACH1 is a novel predictive and prognostic biomarker in hepatocellular carcinoma as a negative regulator of Wnt/beta‐catenin signaling. Oncotarget. 2015;6:8621–8634. 10.18632/oncotarget.3281 25940701 PMC4496171

[52] Perugorria MJ , Olaizola P , Labiano I , Esparza‐Baquer A , Marzioni M , Marin JJG , et al. Wnt–β‐catenin signalling in liver development, health and disease. Nat Rev Gastroenterol Hepatol. 2019;16:121–136. 10.1038/s41575-018-0075-9 30451972

[53] Bennett WFD , Tieleman DP . Molecular simulation of rapid translocation of cholesterol, diacylglycerol, and ceramide in model raft and nonraft membranes. J Lipid Res. 2012;53:421–429. 10.1194/jlr.M022491 22246847 PMC3276465

[54] Bieberich E . Sphingolipids and lipid rafts: novel concepts and methods of analysis. Chem Phys Lipids. 2018;216:114–131. 10.1016/j.chemphyslip.2018.08.003 30194926 PMC6196108

[55] Sezgin E , Levental I , Mayor S , Eggeling C . The mystery of membrane organization: composition, regulation and roles of lipid rafts. Nat Rev Mol Cell Biol. 2017;18:361–374. 10.1038/nrm.2017.16 28356571 PMC5500228

[56] Gammons MV , Renko M , Johnson CM , Rutherford TJ , Bienz M . Wnt signalosome assembly by DEP domain swapping of Dishevelled. Mol Cell. 2016;64:92–104. 10.1016/j.molcel.2016.08.026 27692984 PMC5065529

[57] Brunt L , Scholpp S . The function of endocytosis in Wnt signaling. Cell Mol Life Sci. 2018;75:785–795. 10.1007/s00018-017-2654-2 28913633 PMC5809524

[58] Munthe E , Raiborg C , Stenmark H , Wenzel EM . Clathrin regulates Wnt/β‐catenin signaling by affecting Golgi to plasma membrane transport of transmembrane proteins. J Cell Sci. 2020;133. 10.1242/jcs.244467 32546530

[59] El‐Sayed A , Harashima H . Endocytosis of gene delivery vectors: from clathrin‐dependent to lipid raft‐mediated endocytosis. Mol Ther. 2013;21:1118–1130. 10.1038/mt.2013.54 23587924 PMC3677298

[60] Piacentino ML , Hutchins EJ , Andrews CJ , Bronner ME . Temporal changes in plasma membrane lipid content induce endocytosis to regulate developmental epithelial‐to‐mesenchymal transition. Proc Natl Acad Sci U S A. 2022;119:e2212879119. 10.1073/pnas.2212879119 36508654 PMC9907157

[61] Xia Y , Wei K , Hu LQ , Zhou CR , Lu ZB , Zhan GS , et al. Exosome‐mediated transfer of miR‐1260b promotes cell invasion through Wnt/β‐catenin signaling pathway in lung adenocarcinoma. J Cell Physiol. 2020;235:6843–6853. 10.1002/jcp.29578 32026462

[62] Grammatikos G , Schoell N , Ferreirós N , Bon D , Herrmann E , Farnik H , et al. Serum sphingolipidomic analyses reveal an upregulation of C16‐ceramide and sphingosine‐1‐phosphate in hepatocellular carcinoma. Oncotarget. 2016;7:18095–18105. 10.18632/oncotarget.7741 26933996 PMC4951274

[63] Grbčić P , Car EPM , Sedić M . Targeting ceramide metabolism in hepatocellular carcinoma: new points for therapeutic intervention. Curr Med Chem. 2020;27:6611–6627. 10.2174/0929867326666190911115722 31544710

[64] Krautbauer S , Meier EM , Rein‐Fischboeck L , Pohl R , Weiss TS , Sigruener A , et al. Ceramide and polyunsaturated phospholipids are strongly reduced in human hepatocellular carcinoma. Biochim Biophys Acta. 2016;1861:1767–1774. 10.1016/j.bbalip.2016.08.014 27570113

[65] Bates RC , Fees CP , Holland WL , Winger CC , Batbayar K , Ancar R , et al. Activation of Src and release of intracellular calcium by phosphatidic acid during *Xenopus laevis* fertilization. Dev Biol. 2014;386:165–180. 10.1016/j.ydbio.2013.11.006 24269904 PMC3922219

[66] Myeong J , Park CG , Suh BC , Hille B . Compartmentalization of phosphatidylinositol 4,5‐bisphosphate metabolism into plasma membrane liquid‐ordered/raft domains. Proc Natl Acad Sci U S A. 2021;118:e2025343118. 10.1073/pnas.2025343118 33619111 PMC7936302

[67] Rimmerman N , Hughes HV , Bradshaw HB , Pazos MX , Mackie K , Prieto AL , et al. Compartmentalization of endocannabinoids into lipid rafts in a dorsal root ganglion cell line. Br J Pharmacol. 2008;153:380–389. 10.1038/sj.bjp.0707561 17965731 PMC2219527

[68] Yang Z , Kim S , Mahajan S , Zamani A , Faccio R . Phospholipase Cγ1 (PLCγ1) controls osteoclast numbers via colony‐stimulating factor 1 (CSF‐1)‐dependent diacylglycerol/β‐catenin/CyclinD1 pathway. J Biol Chem. 2017;292:1178–1186. 10.1074/jbc.M116.764928 27941021 PMC5270464

[69] Okada N , Sugiyama K , Shichi S , Shirai Y , Goto K , Sakane F , et al. Combination therapy for hepatocellular carcinoma with diacylglycerol kinase alpha inhibition and anti‐programmed cell death‐1 ligand blockade. Cancer Immunol Immunother. 2022;71:889–903. 10.1007/s00262-021-03041-z 34482409 PMC10991887

[70] Takeishi K , Taketomi A , Shirabe K , Toshima T , Motomura T , Ikegami T , et al. Diacylglycerol kinase alpha enhances hepatocellular carcinoma progression by activation of Ras‐Raf‐MEK‐ERK pathway. J Hepatol. 2012;57:77–83. 10.1016/j.jhep.2012.02.026 22425622

[71] Fazio A , Owusu Obeng E , Rusciano I , Marvi MV , Zoli M , Mongiorgi S , et al. Subcellular localization relevance and cancer‐associated mechanisms of diacylglycerol kinases. Int J Mol Sci. 2020;21:5297. 10.3390/ijms21155297 32722576 PMC7432101

[72] Griner EM , Kazanietz MG . Protein kinase C and other diacylglycerol effectors in cancer. Nat Rev Cancer. 2007;7:281–294. 10.1038/nrc2110 17384583

[73] Beyoğlu D , Idle JR . Metabolomic and lipidomic biomarkers for premalignant liver disease diagnosis and therapy. Metabolites. 2020;10:50. 10.3390/metabo10020050 32012846 PMC7074571

[74] Haraszti RA , Didiot MC , Sapp E , Leszyk J , Shaffer SA , Rockwell HE , et al. High‐resolution proteomic and lipidomic analysis of exosomes and microvesicles from different cell sources. J Extracell Vesicles. 2016;5:32570. 10.3402/jev.v5.32570 27863537 PMC5116062

[75] Hayes CN , Zhang P , Chayama K . The role of lipids in hepatocellular carcinoma. In: Tirnitz‐Parker JEE , editor. Hepatocellular carcinoma. Brisbane: Codon Publications Copyright: The Authors; 2019. 31664805

[76] Mato JM , Alonso C , Noureddin M , Lu SC . Biomarkers and subtypes of deranged lipid metabolism in non‐alcoholic fatty liver disease. World J Gastroenterol. 2019;25:3009–3020. 10.3748/wjg.v25.i24.3009 31293337 PMC6603806

[77] Svegliati‐Baroni G , Pierantonelli I , Torquato P , Marinelli R , Ferreri C , Chatgilialoglu C , et al. Lipidomic biomarkers and mechanisms of lipotoxicity in non‐alcoholic fatty liver disease. Free Radic Biol Med. 2019;144:293–309. 10.1016/j.freeradbiomed.2019.05.029 31152791

[78] Che L , Chi W , Qiao Y , Zhang J , Song X , Liu Y , et al. Cholesterol biosynthesis supports the growth of hepatocarcinoma lesions depleted of fatty acid synthase in mice and humans. Gut. 2020;69:177–186. 10.1136/gutjnl-2018-317581 30954949 PMC6943247

