# Supplementary material for: Comparative membrane lipidomics of hepatocellular carcinoma cells reveals diacylglycerol and ceramide as key regulators of Wnt/β‐catenin signaling and tumor growth
Source: Mol Oncol. 2023 Sep 20;17(11):2314–36. doi: 10.1002/1878-0261.13520 (PMC10620124; doi:10.1002/1878-0261.13520)
Supplement: Supplementary file 1 — Fig. S1. Different HCC cell lines vary in their activity of Wnt/β‐catenin signaling and plasma membrane lipid composition. Fig. S2. Plasma membranes of different HCC cells diverge with respect to distribution of main lipid categories and lipid species. Fig. S3. Membrane lipids of HCC cells alter significantly in response to inhibition of Wnt/β‐catenin signaling. Fig. S4. Global comparison of membrane lipidome profiles reveals differential regulation of lipids in HCC cells and healthy cells. Fig. S5. DAG and ceramide restore Wnt/β‐catenin signaling activity after DGK treatment in SNU475 and HepG2 cells. Fig. S6. DGK reduces membrane DAG in HepG2 and HEK293T cells. Fig. S7. DGKA transfection reduces Wnt/β‐catenin signaling activity in HeLa and HEK293T cells. Fig. S8. Depletion of DAG or ceramide reduces caveolae‐mediated internalization of Wnt‐receptor complex in HepG2 cells. [file MOL2-17-2314-s003.docx]

**Azbazdar et al.**

**Supplementary Figures and Figure Legends**

**
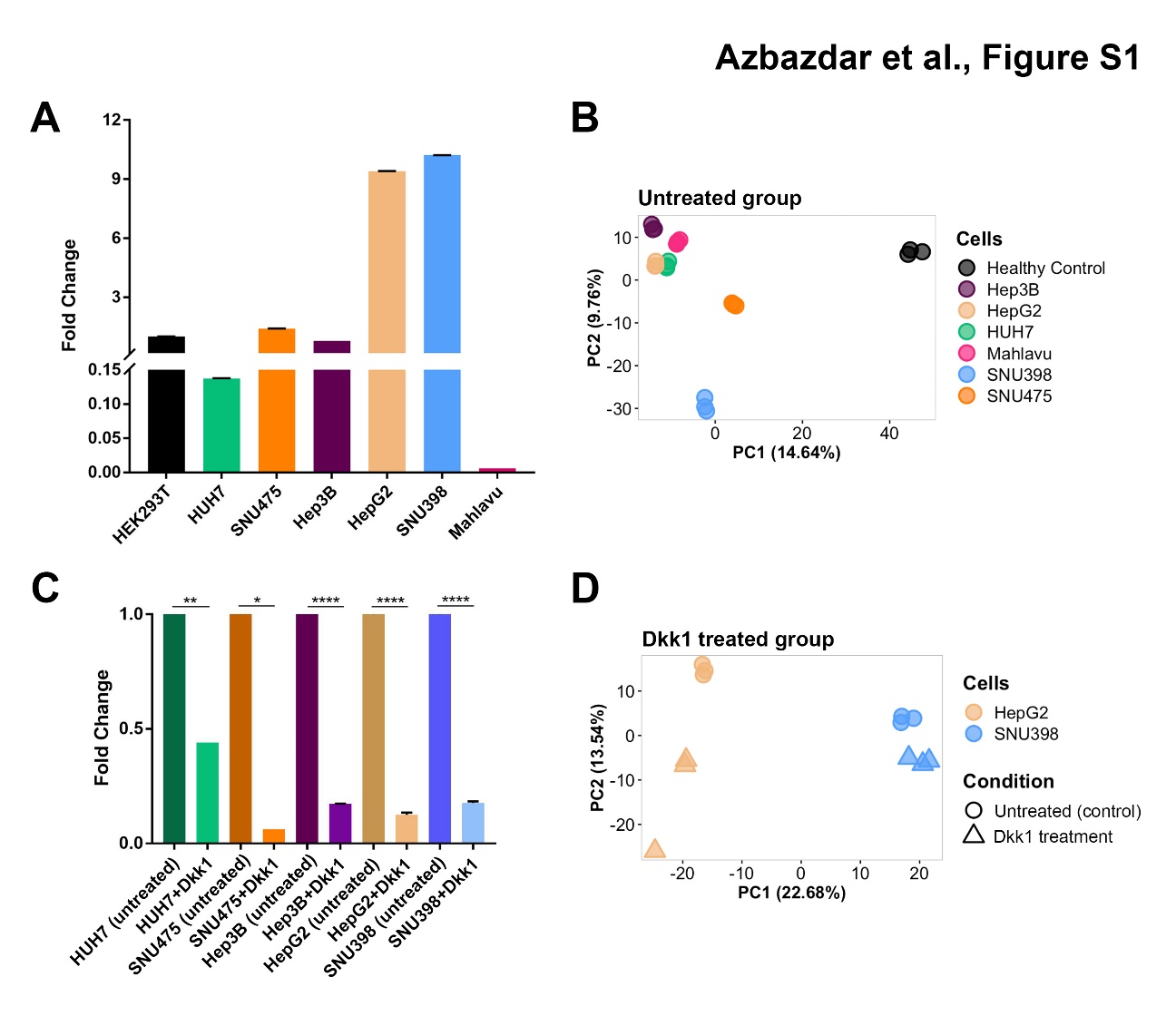
**

**Figure S1: Different HCC cell lines vary in their activity of Wnt/β-catenin signaling and plasma membrane lipid composition.** (A) Comparison of canonical Wnt signaling activity in HEK293T, HUH7, SNU475, Hep3B, HepG2, SNU398 and Mahlavu cells. Average and SD of the mean (error bars) values of pBAR luciferase reporter activity represent Wnt/ß-catenin signaling activity (normalized to renilla luciferase activity) in Statistical significance was evaluated using unpaired t-test. **** p < 0.0001, *** p < 0.001, ** p < 0.01, and * p < 0.05. Error bars represent SD. (B) Principal component analysis (PCA) of untreated healthy control and HCC cells. (C) Comparison of canonical Wnt signaling activity in untreated and Dkk1 treated HUH7, SNU475, Hep3B, HepG2 and SNU398 cells (D) PCA of untreated and Dkk1-treated HepG2 and SNU398 cells. Three independent experiments were performed for (A) and three biological replicates (n=3) were used in each experimental group for the analyses in (B-D).

**
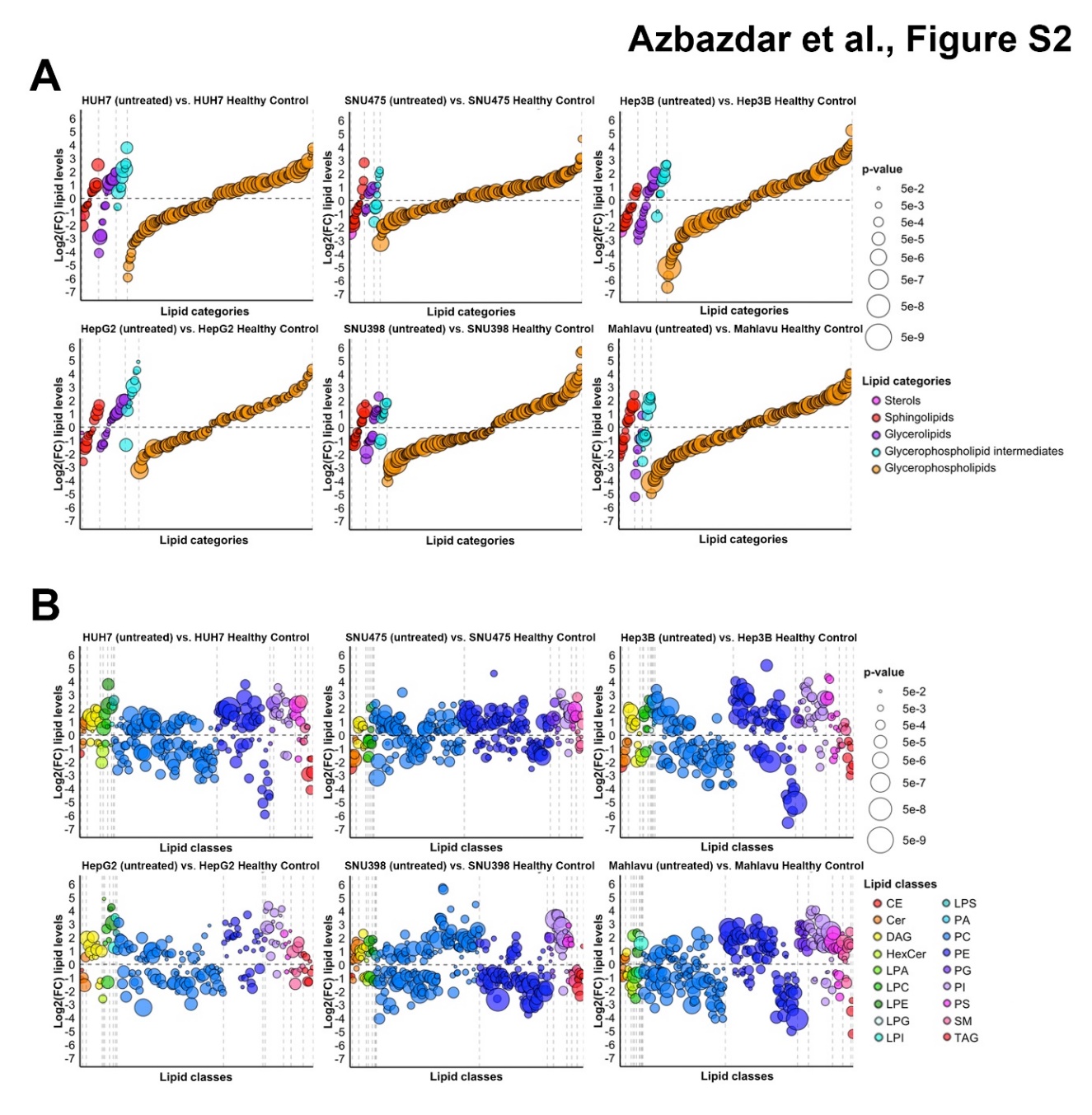
**

**Figure S2:** **Plasma membranes of different HCC cells diverge with respect to distribution of main lipid categories and lipid species.** Differential regulation plots of lipids arranged by (A) lipid categories and (B) lipid classes in untreated HUH7, SNU475, Hep3B, HepG2 and SNU398 cell lines in comparison to healthy control liver cells (THLE2). Lipids are represented by dots arranged regularly on the x-axis with their log2 fold change represented on the y-axis. Dots are colored by (A) lipid category or (B) lipid class and sized proportionally to statistical significance of differential regulation. Only differentially regulated lipid species are shown for each contrast. Vertical dashed lines separate lipid categories/classes. Horizontal dashed line separates up- and down-regulated lipids. Three biological replicates (n=3) were used in each experimental group.

**
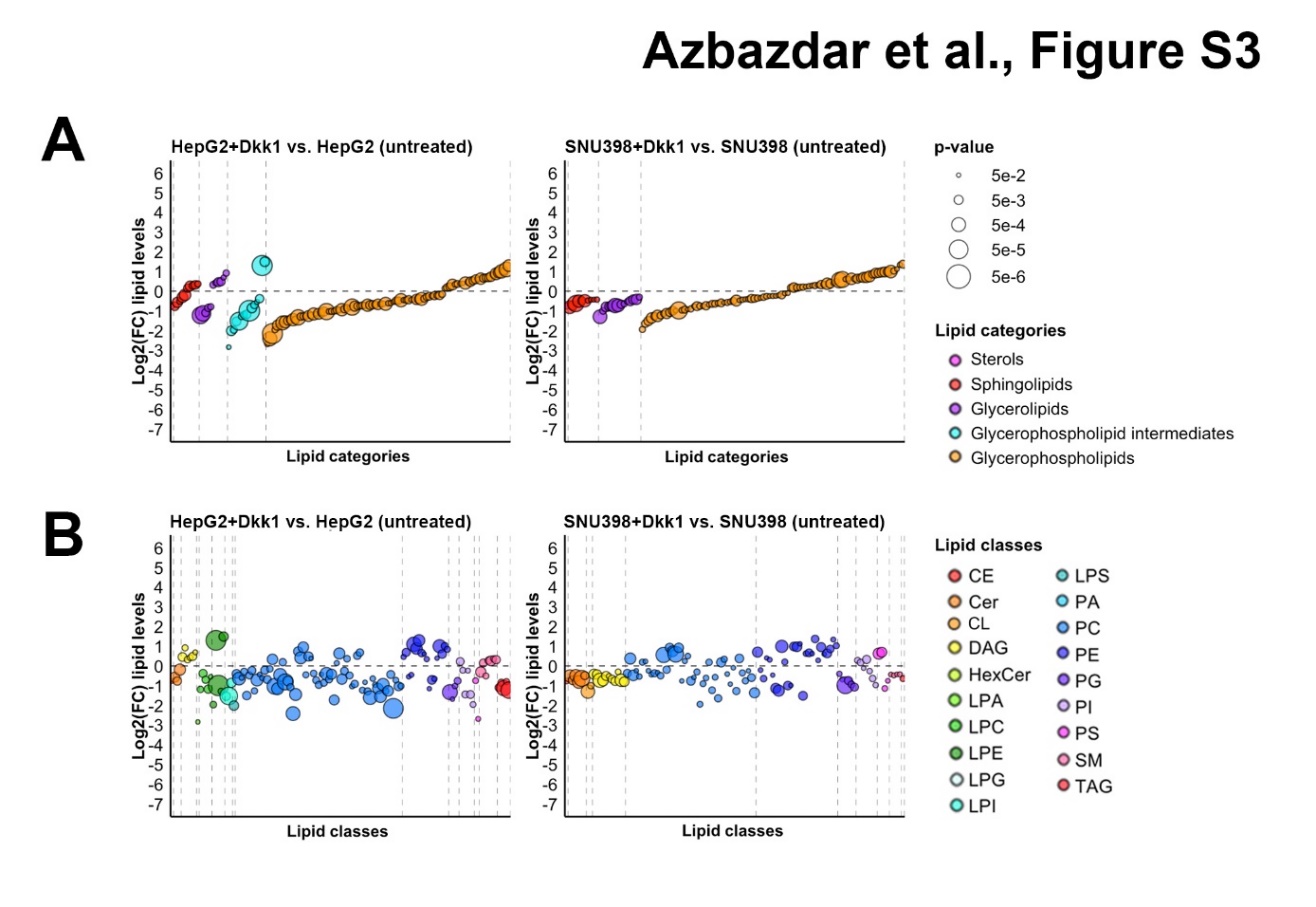
**

**Figure S3: Membrane lipids of HCC cells alter significantly in response to inhibition of Wnt/β-catenin signaling.** Differential regulation plots of lipids in response to Wnt signaling pathway inhibition in selected HCC cell lines. DRLs arranged by (A) lipid categories and (B) lipid classes in response to Dkk1 treatment in HepG2 and SNU398 lines. Lipids are represented by dots arranged regularly on the x-axis with their log2 fold change represented on the y-axis. Dots are colored by (A) lipid category or (B) lipid class and sized proportionally to statistical significance of differential regulation. Only differentially regulated lipid species are shown for each contrast. Vertical dashed lines separate lipid categories/classes. Horizontal dashed line separates up- and down-regulated lipids. Three biological replicates (n=3) were used in each experimental group.


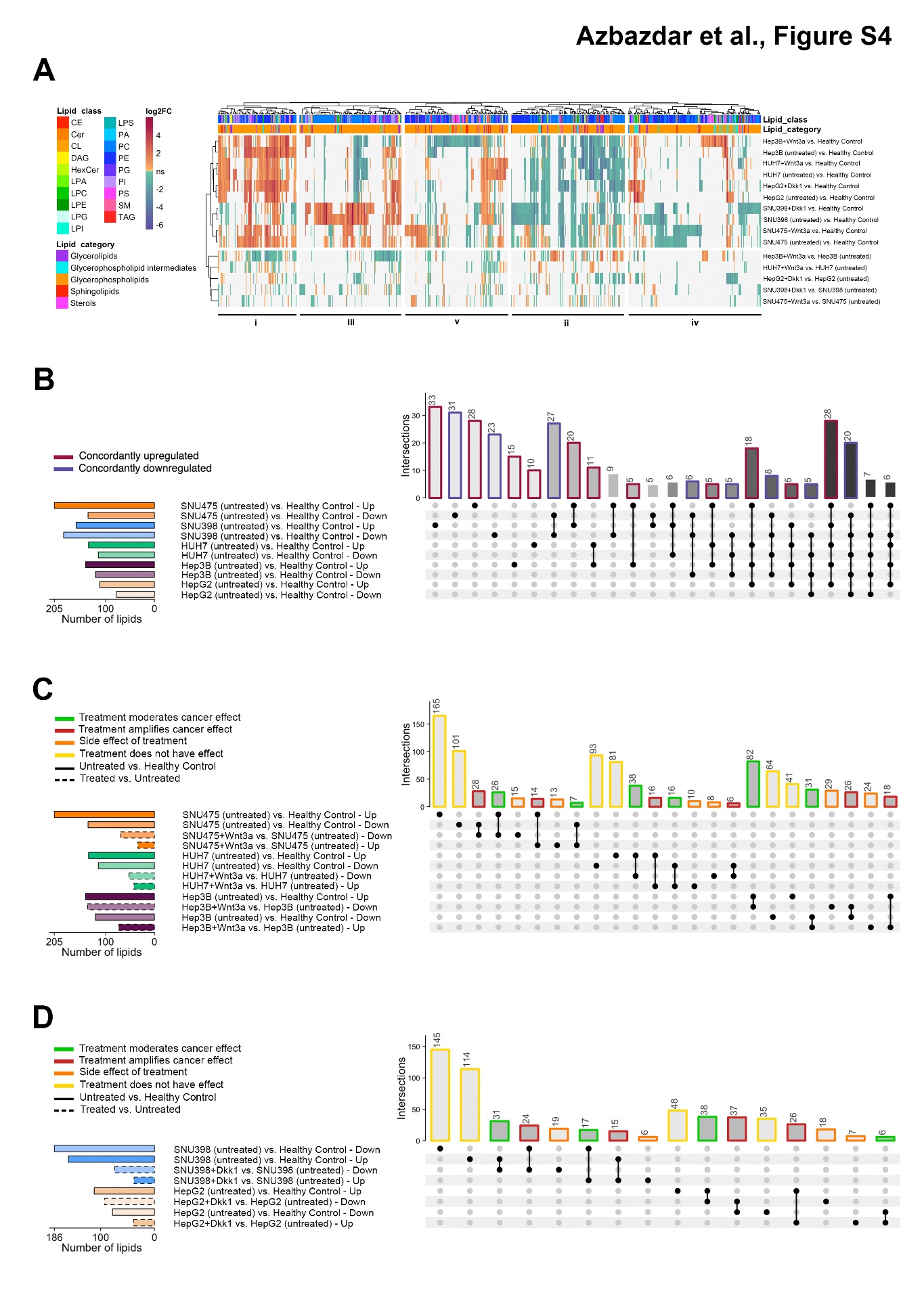


**Figure S4: Global comparison of membrane lipidome profiles reveal differential regulation of lipids in HCC cells and healthy cells.** (A) Heatmap of lipid species and differential regulation contrasts. Contrasts of the following types are all represented by their differential regulation profiles (log2 fold change): untreated HCC vs. healthy control (cancer effect), treated HCC vs. untreated HCC (treatment effect) and treated HCC vs healthy control (global effect of cancer and treatment). Contrasts are clustered horizontally into two main blocks: cancer effect and global effect on the upper block and treatment effect on the lower block. Contrasts are clustered vertically into five main blocks indicated as i-v. (B) UpSet plot showing the commonalities of lipid differential regulation between untreated HCC cell lines. The UpSet plot shows the total numbers of DRLs in each cell line on horizontal bars, separated by direction of regulation (Up or Down), as well as the intersections of these sets of lipids on vertical bars. In red are shown the lipid sets that are concordantly Up and in blue are shown the sets of concordantly Down. Only intersections with more than five lipids are displayed. (C-D) UpSet plots showing the effect of (C) Wnt3a treatment and (D) Dkk1 treatment on lipids, relatively to the effect of cancer on these lipids. Comparisons are drawn only within each cell line. DRLs are split in four classes for each cell line: Green lipids on which the treatment moderates the effect of cancer (i.e. downregulates Up lipids or upregulates Down lipids), red lipids on which the treatment amplifies the effect of cancer (i.e. further upregulates Up lipids or further downregulates Down lipids), yellow lipids that are affected by cancer but on which the treatment does not have any effect, and orange lipids that were not affected by cancer but are differentially regulated by the treatment (side effect). Cancer and treatment related DRL sets are shown by solid and dashed strokes, respectively. Three biological replicates (n=3) were used in each experimental group.

**
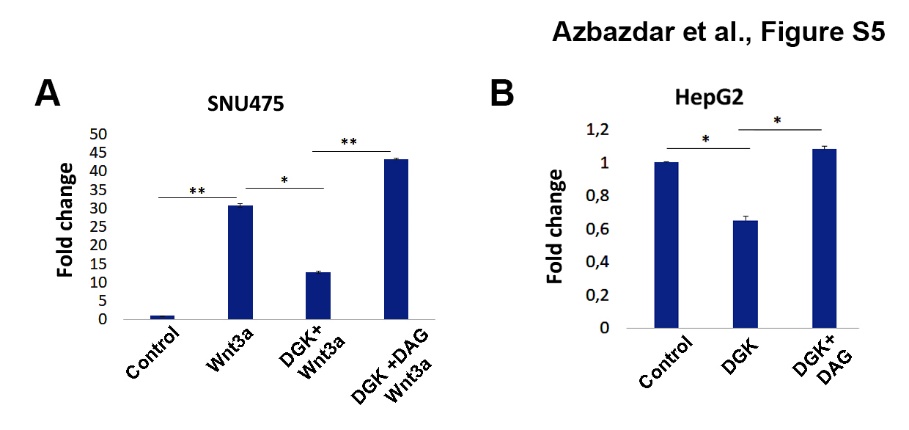
Figure S5:** **DAG and ceramide restore Wnt/β-catenin signaling activity after DGK treatment in SNU475 and HepG2 cells.** Wnt/ß-catenin signaling activity (normalized to renilla luciferase activity) in (A) Wnt3a-treated SNU475 cells treated with DGK or DGK+DAG (B) Dkk1-treated HepG2 cells treated with DGK or DGK+DAG. Average and SD of the mean (error bars) values of pBAR luciferase reporter activity are shown. Statistical significance was evaluated using an unpaired t-test. ** p < 0.01, and * p < 0.05. Error bars represent SD. Three independent experiments were performed.

**
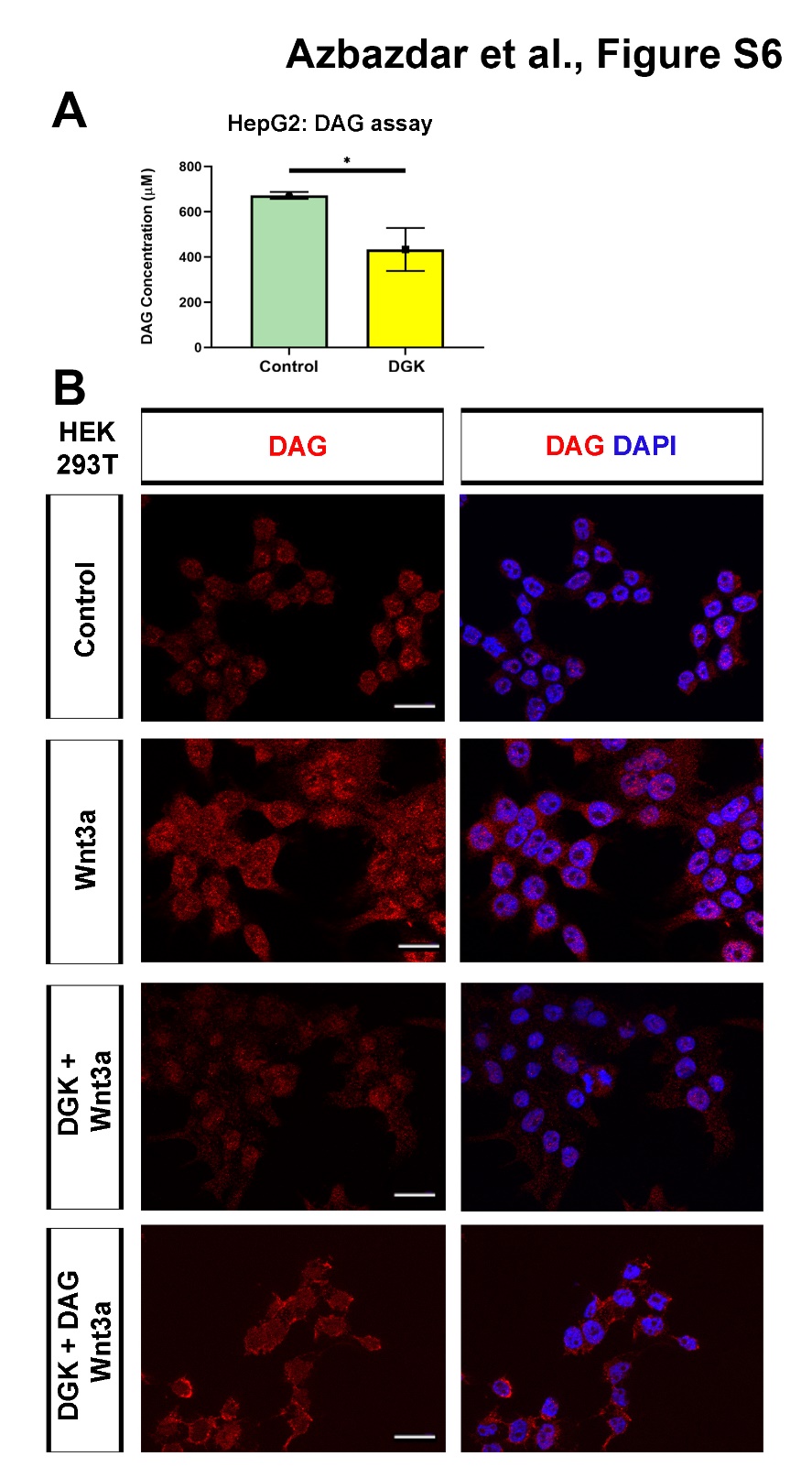
**

**Figure S6:** **DGK reduces membrane DAG in HepG2 and HEK293T cells.** (A) DAG content measured in HepG2 cells treated with DGK. (B) Anti DAG (red) staining of HEK293T cells. Cells are counterstained for DAPI. When compared to Wnt3a-treated cells, DGK addition to Wnt3a-treated cells decreases DAG levels. DAG addition abolishes the suppressor effect of DGK in Wnt3a-treated cells. Statistical significance was evaluated using an unpaired t-test. p < 0.05. Error bars represent standard deviation. Scale bars: 25 µM. Three independent experiments were performed.

**
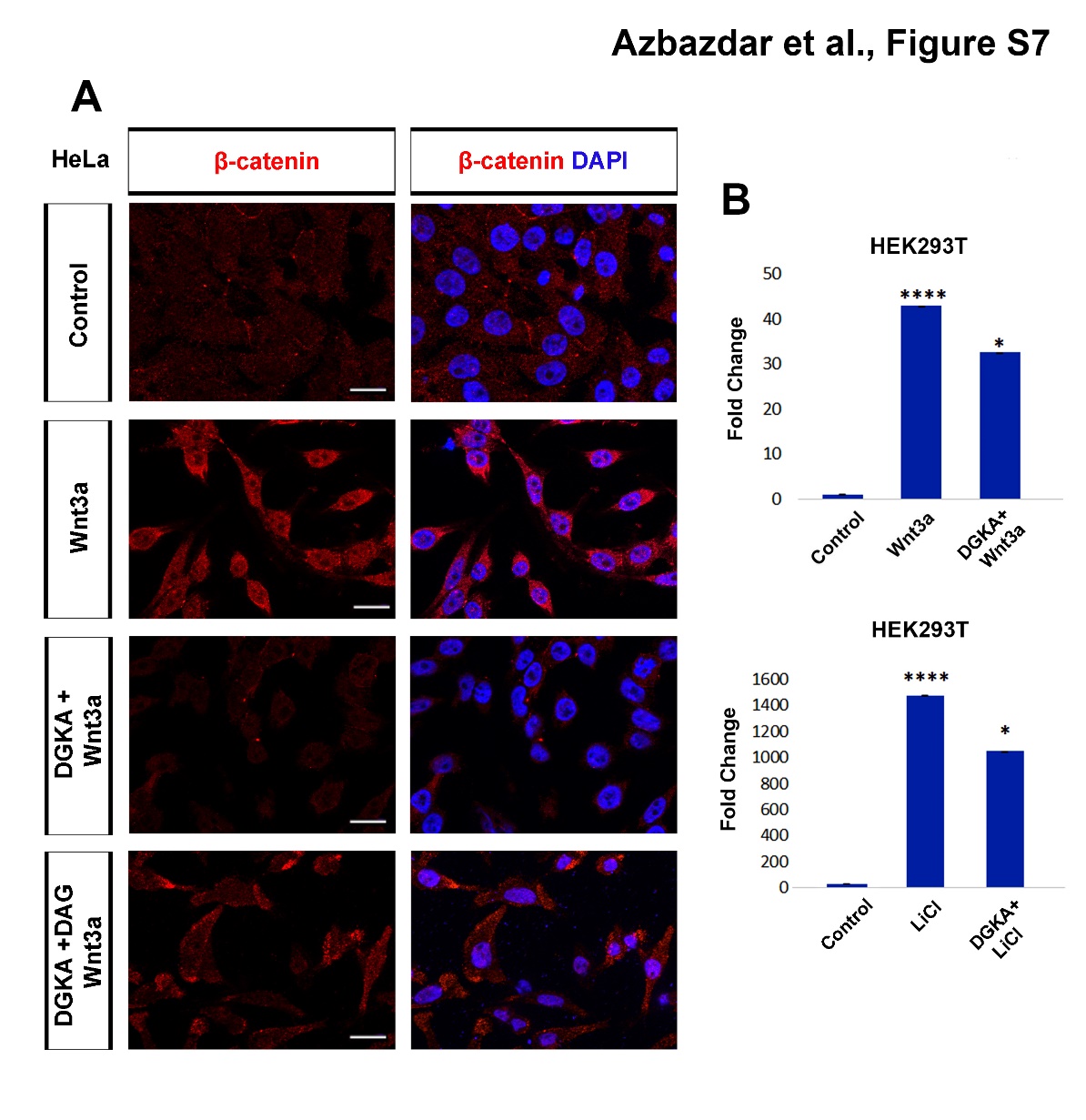
**

**Figure S7:** **DGKA transfection reduces Wnt/β-catenin signaling activity in** **HeLa and HEK293T cells.** (A) Anti β-catenin (red) staining of HeLa cells. Cells are counterstained for DAPI. When compared to Wnt3a-treated cells, DGKA expression in Wnt3a-treated cells decreases expression of β-catenin while DAG addition restores β-catenin levels back to levels detected after Wnt3a treatment. (B) Wnt/ß-catenin signaling activity (normalized to renilla luciferase activity) in (top) Wnt3a-treated and (bottom) LiCl-treated HEK293T cells transfected with DGKA. Average and SD of the mean (error bars) values of pBAR luciferase reporter activity are shown. Statistical significance was evaluated using an unpaired t-test. **** p < 0.0001, and * p < 0.05. Error bars represent SD. Scale bars: 25 µM. Three independent experiments were performed.


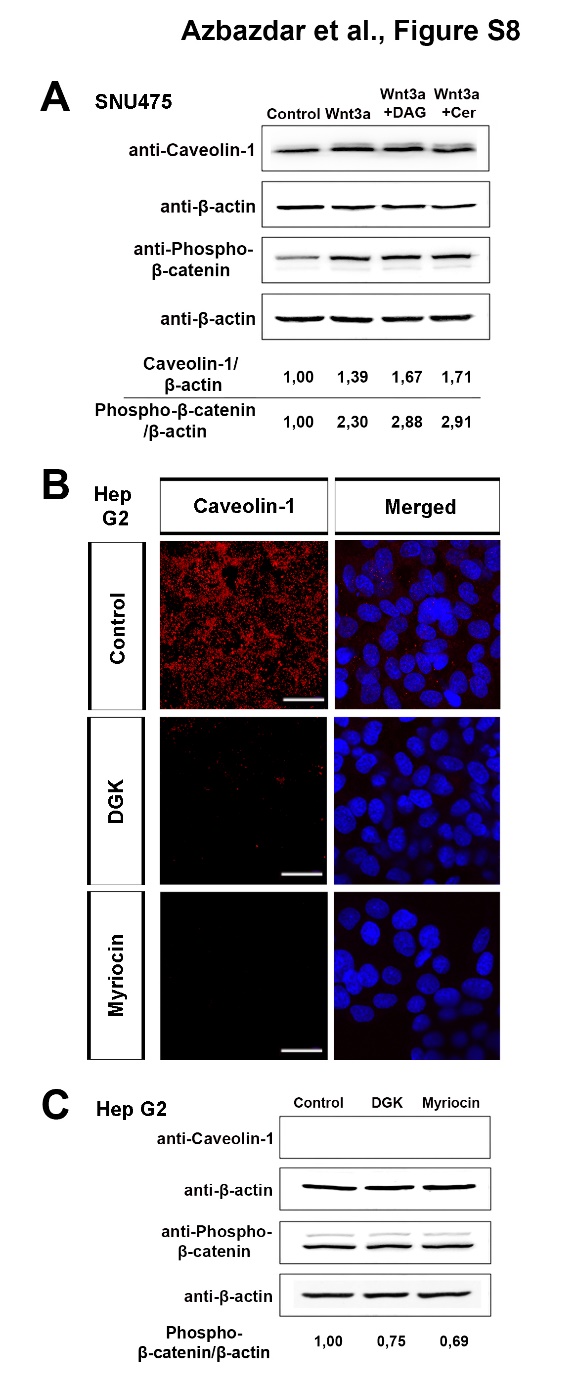


**Figure S8: Depletion of DAG or ceramide reduce caveolae-mediated internalization of Wnt-receptor complex in HepG2 cells.** (A) Western blot of SNU475 cells treated with DAG or ceramide and stimulated with Wnt3a. DAG or ceramide causes an increase in Caveolin-1 levels in cells with activated canonical Wnt signaling, detected by an increase in phospho-β-catenin levels. (B) Anti Caveolin-1 (red) staining of HepG2 cells. Cells are counterstained for DAPI. When compared to Wnt3a-treated cells, treatment of cells with DGK or myriocin reduces expression of Caveolin-1. (C) Western blot of HepG2 cells treated with DGK or myriocin. While DGK or myriocin inhibits canonical Wnt signaling observed by a reduction in phospho-β-catenin, Caveolin-1 is below detectable levels. Scale bars: 25 µM. Three independent experiments were performed.
